# Synthesis of sulfanyl derivatives of 1,2,4-triazoles *via* an acid catalyzed intramolecular cyclization of isothiosemicarbazones: structural characterization, *E*/*Z* isomerism, mechanistic insights and *in vitro* cytotoxicity

**DOI:** 10.1039/d6ra00822d

**Published:** 2026-03-10

**Authors:** Kallivalappil Snisha, Mano Chitra Karthikeyan, Nattamai Bhuvanesh, Antony Joseph Velanganni Arockiam, Ramasamy Karvembu

**Affiliations:** a Department of Chemistry, National Institute of Technology Tiruchirappalli 620 015 India kar@nitt.edu; b Molecular Oncology Laboratory, Department of Biochemistry, School of Life Sciences, Bharathidasan University Tiruchirappalli 620 024 India; c Department of Chemistry, Texas A & M University College Station TX 77842 USA

## Abstract

Sulfanyl derivatives of 1,2,4-triazoles (CL1–CL3) were synthesized *via* a Lewis or Brønsted acid catalyzed intramolecular cyclization of corresponding isothiosemicarbazones (TL1–TL3). All the synthesized isothiosemicarbazones and their cyclized sulfanyl 1,2,4-triazole derivatives were well characterized by spectroscopic techniques and single crystal XRD analyses. The *E*/*Z* isomerism of the isothiosemicarbazones was elucidated using NMR spectroscopy and single crystal XRD analysis. Mechanistic investigations, supported by controlled experiments and spectroscopic evidence, revealed that the cyclization proceeded *via* an ionic pathway with the evolution of hydrogen. The cytotoxic effect of the cyclized sulfanyl 1,2,4-triazole derivatives (CL1–CL3) was evaluated by MTT assay against MDA-MB-231 (breast), MCF-7 (breast), and HeLa (cervical) cancer cell lines, as well as HEK-293 (kidney) normal cell line, taking 5-fluorouracil (5-FU) as a reference drug. All the substituted sulfanyl-1,2,4-triazoles showed higher cytotoxicity toward MDA-MB-231 and HeLa cells than 5-FU, while exhibiting low toxicity toward HEK-293 cells, indicating good selectivity toward cancer cells. Substituted compounds [CL2 (*p*-OCH_3_) and CL3 (*p*-NO_2_)] displayed enhanced activity compared to the unsubstituted one (CL1), indicating the influence of the substituents on cytotoxicity. Fluorescence staining assays (AO/EB, Hoechst 33342, Rhodamine 123 and DCFH-DA) further supported the observed cytotoxic effects, and suggested that the compounds promoted apoptotic cell death *via* intracellular reactive oxygen species (ROS) generation, depletion of mitochondrial membrane potential (MMP) and damage to nuclear material.

## Abstract 2

Sulfanyl derivatives of 1,2,4-triazoles were synthesized *via* a Lewis or Brønsted acid catalyzed intramolecular cyclization of isothiosemicarbazones, and their cytotoxicity was evaluated.

## Graphical abstract

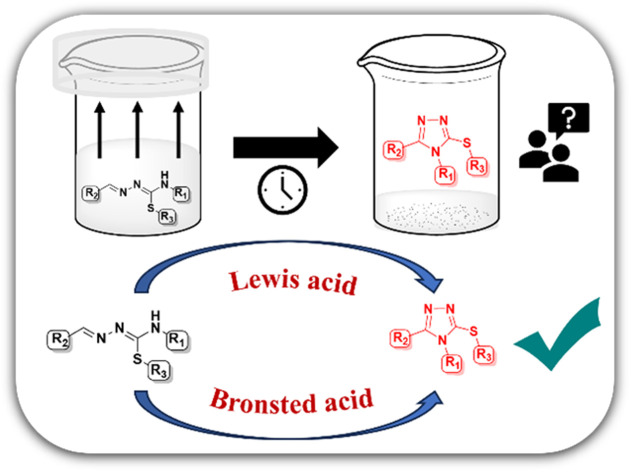

## 1. Introduction

**Fig. 1:**
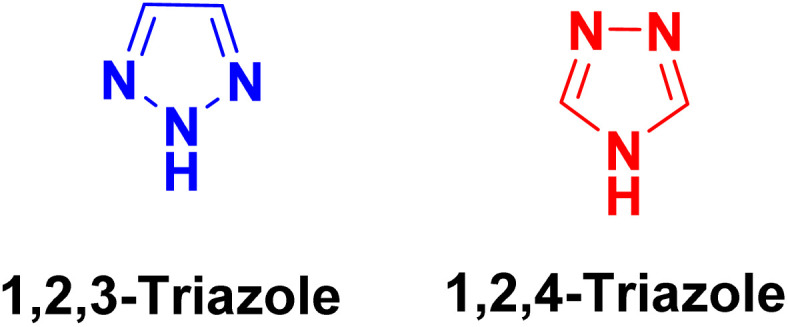
Two isomeric forms of triazole.

**Fig. 2:**
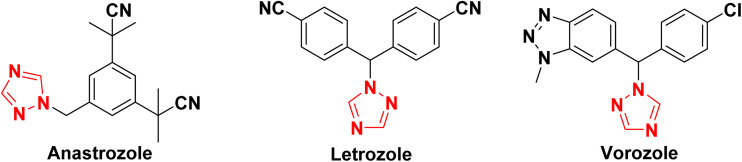
Anticancer drugs containing 1,2,4-triazole nucleus.

Heterocycle-based compounds constitute an important class of molecules in pharmaceutical research owing to their wide spectrum of therapeutic properties.^1,2^ Among these, nitrogen-containing heterocycles, particularly triazoles, have garnered more attention due to their promising biological activities.^1^ Triazoles consist of a five-membered aromatic ring incorporating three nitrogen atoms, and based on the relative positioning of these nitrogen atoms within the ring, triazoles exist in two isomeric forms, namely 1,2,3-triazoles and 1,2,4-triazoles (Fig. 1).^2,3^ Among these, 1,2,4-triazoles^2,4^ are of greater significance due to their well-documented and diverse pharmacological properties, including anticancer,^3,5^ antibacterial,^2^ antifungal,^4,6,7^ antitubercular,^8^ antiviral,^9^ antihypertensive,^10^ antidepressant,^11^ anti-inflammatory,^12^ antimalarial,^13^ antidiabetic,^14^ analgesic^15^ and antimigraine^16^ activities. The pronounced biological efficacy of 1,2,4-triazoles is largely attributed to their rigidity,^7^ stability,^7^ moderate dipole character^7^ and ability to engage in diverse non-covalent interactions^17^ with biological targets. The therapeutic relevance of the 1,2,4-triazole scaffold is further underscored by its presence in several clinically approved drugs such as anastrozole, letrozole and vorozole, which are extensively used in the treatment of hormone-dependent breast cancer^7,18^ (Fig. 2). Other drugs containing a 1,2,4-triazole core are voriconazole, itraconazole, ravuconazole, fluconazole (all antifungal drugs),^2,6,7^ taribavirin (antiviral),^2,9^ rizatriptan (antimigraine),^2,16^ trazodone (antidepressant),^2^*etc*. Sulfanyl derivatives of 1,2,4-triazoles represent a relatively understudied class of heterocycles. The thioether linkage present in these compounds is known to enhance biological activity by increasing aqueous solubility, reducing lipophilicity and providing hydrogen bond acceptor sites.^19,20^ According to previous reports, these derivatives exhibit diverse biological activities such as anticancer,^21^ antimicrobial,^22,23^ antidepressant,^24^ anti-tuberculosis,^25^ antifungal,^23^ antibacterial,^23^ diuretic, neuroleptic and anti-inflammatory effects.^19,26^ The drug ufiprazole, which contains a thioether functionality, is active against cancer.^19^

**Fig. 3:**
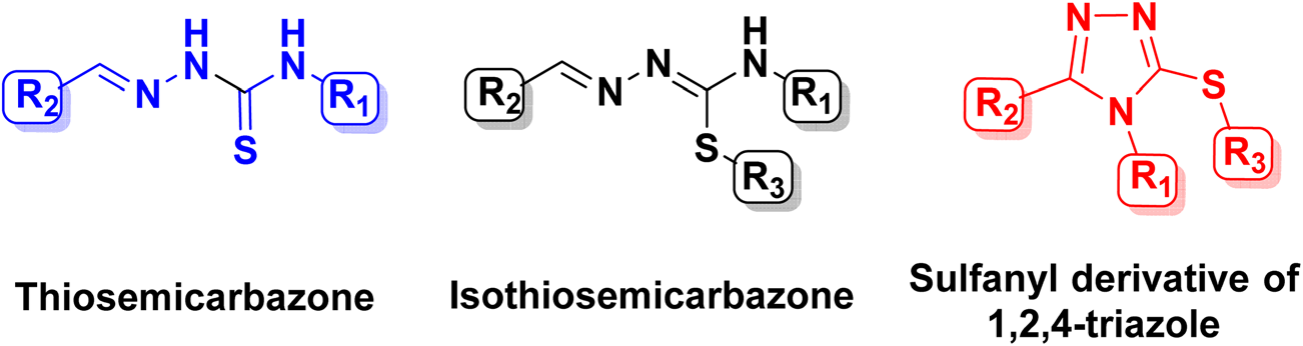
General structures of thiosemicarbazone, isothiosemicarbazone and sulfanyl derivative of 1,2,4-triazole.

There are numerous approaches for the synthesis of 1,2,4-triazole-based compounds from various nitrogen sources like amidines,^27^ imidates,^28^ amidrazones,^29^ aryldiazoniums^30^ and hydrazones.^31^ But there are very few reports available for the synthesis of its sulfanyl derivatives.^19,26,32,33^ Kaldrikyan *et al.* reported the synthesis of substituted sulfanyl-1,2,4-triazoles starting from thiosemicarbazide, involving cyclization to form the 1,2,4-triazole core followed by *S*-alkylation.^34^ The synthesis of sulfanyl derivatives of 1,2,4-triazole from isothiosemicarbazone was found to be quite interesting, and to the best of our knowledge, there is no report for the acid-catalyzed cyclization of isothiosemicarbazones into sulfanyl derivatives of 1,2,4-triazole. Isothiosemicarbazones are a class of Schiff base compounds,^35^ which have been less explored.^36^ Isothiosemicarbazones are the enol form of classical thiosemicarbazones,^36^ and the reactions of thiosemicarbazones with alkyl/aryl halide (RX/ArX) in a suitable solvent yield isothiosemicarbazones incorporated with HX.^35,37,38^ Structurally, isothiosemicarbazones are prone to *E*/*Z* isomerism^38^ around the C=N bond, often resulting in inseparable isomeric mixtures in solution.^35^ This inherent isomerism poses a significant challenge for their biological evaluation, as the presence of multiple interconverting species complicates the establishment of reliable structure–activity relationships. In the course of recrystallization of isothiosemicarbazone derivatives (HX form), an unexpected cyclization was observed under the employed conditions, leading to the formation of crystals of sulfanyl-1,2,4-triazole derivatives rather than those of isothiosemicarbazones (Fig. 3). This drove us to conduct a deeper research into the formation of sulfanyl derivatives of 1,2,4-triazoles from isothiosemicarbazones.

Given the established relevance of heterocycles, particularly nitrogen-containing systems in anticancer drug discovery, the incidental formation of stable sulfanyl derivatives of 1,2,4-triazole from isothiosemicarbazones motivated further exploration. Tuning of a compound by the introduction of a new moiety, varying the substituent and position of the substituent, also significantly matters in medicinal chemistry. Based on these considerations, the present work describes the synthesis of a series of isothiosemicarbazones and their cyclized sulfanyl 1,2,4-triazole derivatives bearing electron withdrawing or electron donating substituent, with the aim of elucidating the influence of electronic effects on their cytotoxicity. Cyclized sulfanyl 1,2,4-triazole derivatives were synthesized by the Lewis or Brønsted acid catalyzed intramolecular cyclization of isothiosemicarbazones. Isothiosemicarbazones are comparatively less explored; *E*/*Z* isomerism exhibited by isothiosemicarbazones was studied in detail using NMR spectroscopy and single crystal XRD. A good attempt was made to explore the mechanism of acid-catalyzed intramolecular cyclization of isothiosemicarbazones with some controlled experiments and spectroscopic techniques. Cytotoxicity of cyclized sulfanyl 1,2,4-triazole compounds was evaluated by MTT assay and fluorescence staining assays against cancer cell lines such as MDA-MB-231, MCF-7 and HeLa, which were selected in consideration of their high global prevalence.^39^

## 2. Experimental section

### 2.1. Materials and methods

All the chemicals used in this work were purchased from Sigma Aldrich, Alfa-Aesar or Avra. The melting points were determined using a Lab India instrument and are uncorrected. Fourier transform-infrared (FT-IR) spectra were recorded with a Thermo Scientific Nicolet iS5 FT-IR spectrometer by using KBr pellets of the compounds. Ultraviolet-visible (UV-vis) spectra of the compounds were recorded using a Shimadzu UV-2600 instrument. Nuclear magnetic resonance (NMR) spectra were recorded in dimethyl sulfoxide-*d*_6_ (DMSO-*d*_6_)/CDCl_3_/CD_3_OD using tetramethylsilane (TMS) as an internal standard on a Bruker 500 MHz spectrometer. High resolution mass spectra (HRMS) were recorded with an Agilent QTOF G6545 XT spectrometer at 50 000 resolutions using ESI mode.

### 2.2. Synthesis

#### 2.2.1. Synthesis of isothiosemicarbazones (TL1–TL3)

To an ethanolic solution (5 mL) of 4-methyl-3-thiosemicarbazide (0.2628 g, 2 mmol), an ethanolic solution (5 mL) of benzyl bromide (0.2973 mL, 2.2 mmol) was added dropwise with constant stirring. The resulting mixture was stirred for 16 h under reflux at 80 °C. To the above reaction mixture, (un)substituted benzaldehyde [0.2551 mL (benzaldehyde), 0.3039 mL (*p*-methoxybenzaldehyde) or 0.3778 g (*p-*nitrobenzaldehyde), 2.5 mmol] in ethanol (5 mL) was added dropwise, and the stirring was continued under reflux for another 6–12 h at 80 °C. The completion of the reaction was monitored by thin layer chromatography (TLC). The reaction mixture was kept in a deep freezer overnight to obtain the precipitate in good yield. The precipitate was filtered and washed with cold ethanol to remove any unreacted reactants, followed by cold diethyl ether to eliminate residual organic impurities [yield: 77% (TL1·HBr), 74% (TL2·HBr) and 83% (TL3·HBr)]. The obtained precipitate was worked up with dichloromethane-water mixture, and the dichloromethane layer was evaporated under reduced pressure using rotavapor and dried under *vacuum*. TL1 was recrystallized from its saturated ethanolic solution, and TL3 was recrystallized from its saturated solution of acetonitrile and a few drops of methanol.

##### 2.2.1.1. Benzyl-*N*′-benzylidene-*N*-methylcarbamohydrazonothioate (TL1)

Yield: 74%. Off white solid. M.p.: 96 °C. FT-IR [KBr, *ν* (cm^−1^)]: 3441 (N–H), 1606 (C^1^=N^1^), 1570 (C^2^=N^2^), 1161 (N^1^–N^2^), 752 (C^2^–S^1^). UV-vis [CH_3_CN, *λ*_max_ (nm)]: 232 (π → π*), 318 (n → π*). ^1^H NMR [500 MHz, DMSO-*d*_6_, *δ* (ppm)]: 8.22 (s, 1H, imine CH), 7.64 (d, *J* = 8.3 Hz, 2H, aromatic CH), 7.41 (d, *J* = 7.1 Hz, 2H, aromatic CH), 7.38–7.32 (m, 5H, aromatic CH), 7.31 (d, *J* = 6.5 Hz, 1H, aromatic CH), 6.69 (s, 1H, exchangeable with D_2_O, NH), 4.18 (s, 2H, benzylic CH_2_), 2.87 (d, *J* = 4.5 Hz, 3H, terminal NCH_3_). ^13^C NMR [126 MHz, DMSO-*d*_6_, *δ* (ppm)]: 162.98 (C^2^=N^2^), 150.80 (C^1^=N^1^), 137.16, 136.12, 129.61, 129.57, 129.10, 129.04, 128.94, 128.85, 127.96, 127.77, 127.58, 127.28 (aromatic carbons), 33.87 (benzylic CH_2_), 30.77 (terminal NCH_3_). HRMS (*m*/*z*): found (calcd.) 284.1229 (284.1221) {[M + H]^+^ = [C_16_H_18_N_3_S]^+^}.

##### 2.2.1.2. Benzyl-*N*′-(4-methoxybenzylidene)-*N*-methylcarbamohydrazonothioate (TL2)

Yield: 72%. Off white solid. M.p.: 118 °C. FT-IR [KBr, *ν* (cm^−1^)]: 3373 (N–H), 1601 (C^1^=N^1^), 1509 (C^2^=N^2^), 1166 (N^1^–N^2^), 714 (C^2^–S^1^), 2926 (aliphatic C–H of OCH_3_), 1245 (asymmetric C–O–C stretching), 1029 (symmetric C–O–C stretching). UV-vis [CH_3_CN, *λ*_max_ (nm)]: 257 (π → π*), 332 (n → π*). ^1^H NMR [500 MHz, DMSO-*d*_6_, *δ* (ppm)]: 8.16 (s, 1H, imine CH), 7.58 (d, *J* = 8.8 Hz, 2H, aromatic CH), 7.40 (d, *J* = 7.0 Hz, 2H, aromatic CH), 7.36 (t, *J* = 7.5 Hz, 2H, aromatic CH), 7.30 (d, *J* = 7.2 Hz, 1H, aromatic CH), 6.94 (d, *J* = 8.8 Hz, 2H, aromatic CH), 6.56 (q, *J* = 4.3 Hz, 1H, exchangeable with D_2_O, NH), 4.17 (s, 2H, benzylic CH_2_), 3.77 (s, 3H, OCH_3_), 2.84 (d, *J* = 4.5 Hz, 3H, terminal NCH_3_). ^13^C NMR [126 MHz, DMSO-*d*_6_, *δ* (ppm)]: 161.95 (C^2^=N^2^), 160.66 (C^1^=N^1^), 150.64, 137.27, 129.59, 129.50, 129.08, 128.84, 128.79, 127.73, 114.57 (aromatic carbons), 55.67 (OCH_3_), 33.85 (benzylic CH_2_), 30.75 (terminal NCH_3_). HRMS (*m*/*z*): found (calcd.) 314.1326 (314.1327) {[M + H]^+^ = [C_17_H_20_N_3_OS]^+^}.

##### 2.2.1.3. Benzyl-*N*-methyl-*N*′-(4-nitrobenzylidene)carbamohydrazonothioate (TL3)

Yield: 78%. Orange solid. M.p.: 162 °C. FT-IR [KBr, *ν* (cm^−1^)]: 3434 (N–H), 1592 (C^1^=N^1^), 1519 (C^2^=N^2^), 1168 (N^1^–N^2^), 765 (C^2^–S^1^), 1497 (asymmetric NO_2_ vibration), 1328 (symmetric NO_2_ vibration). UV-vis [CH_3_CN, *λ*_max_ (nm)]: 215 (π → π*), 250 (n → π*), 382 (n → π*). ^1^H NMR [500 MHz, DMSO-*d*_6_, *δ* (ppm)]: 8.33 (s, 1H, imine CH), 8.23 (d, *J* = 8.9 Hz, 2H, aromatic CH), 7.87 (d, *J* = 8.9 Hz, 2H, aromatic CH), 7.45–7.40 (m, 2H, aromatic CH), 7.37 (t, *J* = 7.5 Hz, 2H, aromatic CH), 7.33–7.30 (m, 1H, aromatic CH), 7.04 (q, *J* = 4.5 Hz, 1H, exchangeable with D_2_O, NH), 4.21 (s, 2H, benzylic CH_2_), 2.91 (d, *J* = 4.5 Hz, 3H, terminal NCH_3_). ^13^C NMR [126 MHz, DMSO-*d*_6_, *δ* (ppm)]: 165.26 (C^2^=N^2^), 148.35 (C^1^=N^1^), 147.58, 142.64, 136.78, 129.68, 129.63, 129.16, 128.89, 128.59, 127.87, 124.46, 124.20 (aromatic carbons), 33.89 (benzylic CH_2_), 30.88 (terminal NCH_3_). HRMS (*m*/*z*): found (calcd.) 329.1072 (329.1072) {[M + H]^+^ = [C_16_H_17_N_4_O_2_S]^+^}.

#### 2.2.2. Synthesis of sulfanyl derivatives of 1,2,4-triazoles (CL1–CL3)

Isothiosemicarbazone (hydrobromide salt) (200 mg) was dissolved in methanol (10 mL) and kept under reflux for 8–12 h at 60 °C. Solvent in the reaction mixture was evaporated by rotavapor, and the crude was purified by column chromatography (hexane-ethyl acetate). All the cyclized sulfanyl 1,2,4-triazole derivatives were crystallized from their saturated solution of methanol by the slow evaporation method.

##### 2.2.2.1. 3-(Benzylthio)-4-methyl-5-phenyl-4H-1,2,4-triazole (CL1)

Yield: 75%. Off white solid. M.p.: 134 °C. FT-IR [KBr, *ν* (cm^−1^)]: 1467 (C=N), 1148 (N–N), 775 (C^2^–S^1^). UV-vis [CH_3_CN, *λ*_max_ (nm)]: 219, 252 (π → π*). ^1^H NMR [500 MHz, CDCl_3_, *δ* (ppm)]: 7.51–7.47 (m, 2H, aromatic CH), 7.44–7.40 (m, 2H, aromatic CH), 7.25–7.20 (m, 5H, aromatic CH), 4.32 (s, 2H, benzylic CH_2_), 3.20 (s, 3H, terminal NCH_3_). ^13^C NMR [126 MHz, CDCl_3_, *δ* (ppm)]: 156.05 (C^2^=N^2^), 150.91 (C^1^=N^1^), 136.97, 130.13, 129.09, 128.91, 128.71, 128.59, 127.83, 127.15 (aromatic carbons), 38.98 (benzylic CH_2_), 31.49 (NCH_3_). HRMS (*m*/*z*): found (calcd.) 282.1067 (282.1065) {[M + H]^+^ = [C_16_H_16_N_3_S]^+^}.

##### 2.2.2.2. 3-(Benzylthio)-5-(4-methoxyphenyl)-4-methyl-4H-1,2,4-triazole (CL2)

Yield: 73%. Off white solid. M.p.: 128 °C. FT-IR [KBr, *ν* (cm^−1^)]: 1468 (C=N), 1175 (N–N), 700 (C^2^–S^1^), 2922 (aliphatic C–H of OCH_3_), 1247 (asymmetric C–O–C stretching), 1033 (symmetric C–O–C stretching). UV-vis [CH_3_CN, *λ*_max_ (nm)]: 212, 258 (π → π*). ^1^H NMR [500 MHz, DMSO-*d*_6_, *δ* (ppm)]: 7.62 (d, *J* = 8.9 Hz, 2H, aromatic CH), 7.36–7.26 (m, 5H, aromatic CH), 7.12 (d, *J* = 11.8 Hz, 2H, aromatic CH), 4.38 (s, 2H, benzylic CH_2_), 3.84 (s, 3H, OCH_3_), 3.42 (s, 3H, NCH_3_). ^13^C NMR [126 MHz, DMSO-*d*_6_, *δ* (ppm)]: 161.14 (C^2^=N^2^), 155.53 (C^2^=N^2^), 150.44, 137.62, 130.45, 129.46, 128.97, 128.02, 119.17, 114.87 (aromatic carbons), 55.85 (OCH_3_), 37.85 (benzylic CH_2_), 32.10 (NCH_3_). HRMS (*m*/*z*): found (calcd.) 312.1172 (312.1171) {[M + H]^+^ = [C_17_H_18_N_3_OS]^+^}.

##### 2.2.2.3. 3-(Benzylthio)-4-methyl-5-(4-nitrophenyl)-4H-1,2,4-triazole (CL3)

Yield: 79%. yellow solid. M.p.: 168 °C. FT-IR [KBr, *ν* (cm^−1^)]: 1467 (C=N), 1139 (N–N), 774 (C^2^–S^1^), 1512 (asymmetric NO_2_ vibration), 1343 (symmetric NO_2_ vibration). UV-vis [CH_3_CN, *λ*_max_ (nm)]: 203, 222 (π → π*), 308 (n → π*). ^1^H NMR [500 MHz, DMSO-*d*_6_, *δ* (ppm)]: 8.39 (d, *J* = 8.9 Hz, 2H, aromatic CH), 8.01 (d, *J* = 8.9 Hz, 2H, aromatic CH), 7.38 (d, *J* = 6.9 Hz, 2H, aromatic CH), 7.35–7.28 (m, 3H, aromatic CH), 4.43 (s, 2H, benzylic CH_2_), 3.53 (s, 3H, terminal NCH_3_). ^13^C NMR [126 MHz, DMSO-*d*_6_, *δ* (ppm)]: 154.25 (C^2^=N^2^), 151.99 (C^1^=N^1^), 148.53, 137.59, 133.61, 129.95, 129.48, 128.98, 128.05, 124.53 (aromatic carbons), 37.58 (benzylic CH_2_), 32.36 (NCH_3_). HRMS (*m*/*z*): found (calcd.) 327.0918 (327.0916) {[M + H]^+^ = [C_16_H_15_N_4_O_2_S]^+^}.

### 2.3. Single crystal X-ray diffraction (XRD) analyses

A suitable crystal was selected and mounted on a MITIGEN holder on a XtaLAB Synergy, Dualflex, HyPix diffractometer. The crystal was kept at a steady *T* = 100.00(10) K during data collection. The structure was solved with the ShelXT 2018/2 solution program using dual methods and by using Olex2 1.5 as the graphical interface.^40,41^ The model was refined with ShelXL 2019/1 using full matrix least squares minimization on *F*^2^.^42^

### 2.4. Solution stability

Solution stability of the cyclized sulfanyl 1,2,4-triazoles (CL1–CL3) was evaluated in different media, like DMSO, 1 : 99 (v/v) DMSO-water, and phosphate-buffered saline (PBS) solution (pH = 7.4) over a period of 24 h using UV-vis spectroscopy.^43^ Stock solutions of the compounds were prepared in DMSO at a concentration of 3 × 10^−3^ M. From each stock solution, 10 µL was added into a cuvette containing 2990 µL of either DMSO, water or PBS, and UV-vis spectra were recorded immediately (0 min) and subsequently at 2 min, 5 min, 15 min, 1 h, 4 h, 8 h and 24 h in all the media at room temperature.

### 2.5. *In vitro* cytotoxicity

#### 2.5.1. Cell culture and statistical analysis

The normal HEK-293 cell line, breast cancer cell lines MDA-MB-231 and MCF-7, and the cervical cancer cell line HeLa were obtained from the National Centre for Cell Science (NCCS), Pune, India. Cells were maintained in Dulbecco's modified eagle's medium (DMEM) supplemented with 10% fetal bovine serum (FBS) and penicillin-streptomycin (Invitrogen, Carlsbad, CA, USA). All cultures were incubated at 37 °C in a humidified atmosphere containing 5% CO_2_. Statistical analysis was carried out using GraphPad Prism (version 9.5.0; GraphPad Software, Inc). Experimental results are expressed as mean ± standard deviation (SD). Comparisons between multiple groups were performed using one-way analysis of variance (ANOVA), and differences were considered statistically significant when *p* < 0.05.^44^

#### 2.5.2. Cytotoxicity assessment

Cell viability following treatment was determined using the MTT [3-(4,5-dimethylthiazol-2-yl)-2,5-diphenyltetrazolium bromide] assay.^44^ Cells were seeded into 96-well culture plates at a density of 1 × 10^4^ cells per well and allowed to adhere overnight under standard culture conditions (37 °C, 5% CO_2_). Cells were treated with varying concentrations of CL1–CL3 and 5-fluorouracil (5-FU), and incubated for 24 h. Subsequently, MTT solution (5 mg mL^−1^ prepared in PBS) was added and incubated for 3–4 h at 37 °C to facilitate the formation of insoluble formazan crystals. The formazan crystals formed were solubilized using DMSO. Absorbance was measured at 570 nm using a microplate reader (Bio-Rad, USA). Cell viability was calculated as a percentage relative to untreated control cells. The half-maximal inhibitory concentration (IC_50_) values were obtained using GraphPad Prism software. The experiments were conducted in triplicate.

#### 2.5.3. Acridine orange/ethidium bromide (AO/EB) dual staining

To examine apoptosis-related morphological alterations, cells were seeded in six-well plates and treated with CL1–CL3 (IC_50_ concentration) for 24 h. Following treatment, the culture medium was discarded, and cells were gently rinsed with PBS. Cells were then stained with AO/EB dye (100 µg mL^−1^) for 5 min at room temperature. Apoptotic features were visualized using a fluorescence microscope (Floid cell imaging station).^45^

#### 2.5.4. Hoechst 33342 nuclear staining

Apoptotic nuclear changes (nuclear fragmentation and chromatin condensation) were assessed using Hoechst 33342 staining. Cells were grown in six-well plates and treated with CL1–CL3 for 24 h. Following treatment, the cells were incubated with 500 µL Hoechst 33342 (10 µg mL^−1^) dye for 30 min in the dark. PBS wash was done to remove excess stain, and nuclear morphology was examined using a fluorescence microscope at 20× magnification.^45^

#### 2.5.5. Rhodamine 123 (Rh-123) staining

Mitochondrial membrane potential (MMP) changes were assessed using Rh-123, a mitochondria-specific fluorescent probe. Cancer cells were treated with CL1–CL3 for 24 h, washed with PBS (pH 7.4), cells were then incubated with 5 µg mL^−1^ Rh-123 for 30 min at 37 °C. After washing with PBS, fluorescence signals were observed using a fluorescence microscope to evaluate mitochondrial integrity.^45^

#### 2.5.6. Detection of intracellular reactive oxygen species (ROS)

Intracellular ROS generation was measured using DCFH-DA (2′,7′-dichlorodihydrofluorescein diacetate) staining. Cells cultured in six-well plates were treated with CL1–CL3 for 24 h. After incubation, the medium was removed, and cells were washed with PBS followed by staining with 100 µM DCFH-DA for 30 min at 37 °C in the dark. Fluorescence intensity, indicating ROS accumulation, was captured using a cell imaging system.^45^

## 3. Results and discussion

### 3.1. Synthesis

**Scheme 1:**
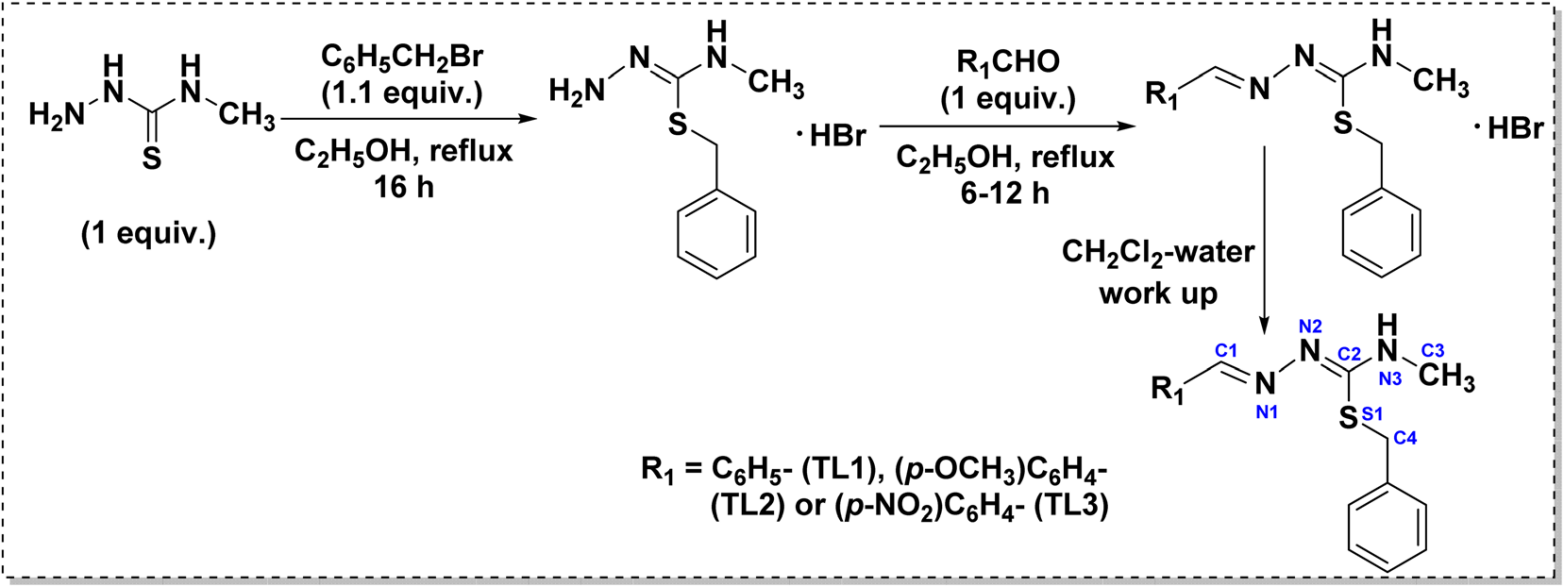
Synthesis of isothiosemicarbazones (TL1–TL3).

**Scheme 2:**
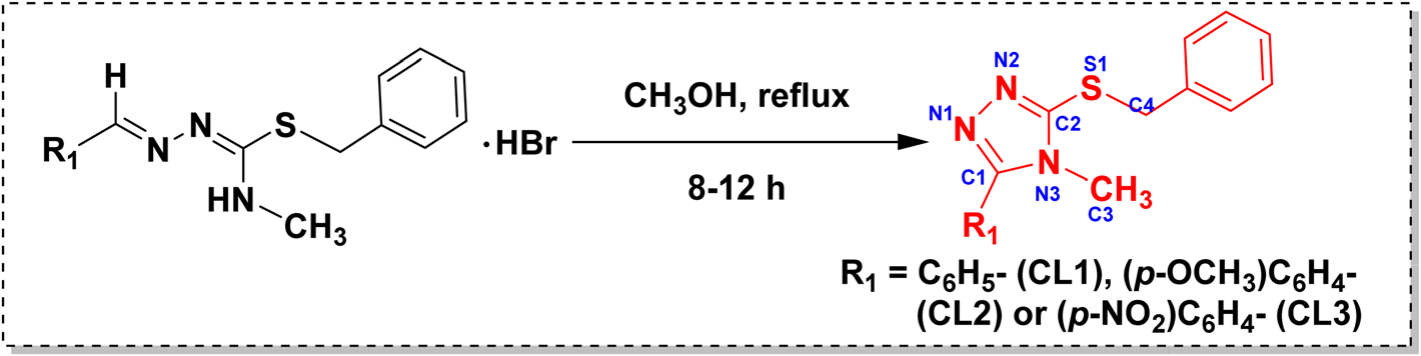
Synthesis of isothiosemicarbazone-based sulfanyl derivatives of 1,2,4-triazoles (CL1–CL3).

The syntheses of the isothiosemicarbazones and their cyclized sulfanyl 1,2,4-triazole derivatives are depicted in Scheme 1 and 2, respectively. The isothiosemicarbazones (TL1–TL3) were synthesized according to a procedure reported in the literature.^35^ Initially, *N*^4^-substituted thiosemicarbazide was treated with benzyl bromide in ethanol solvent under reflux condition to get isothiosemicarbazide. All the isothiosemicarbazones (TL1–TL3) were obtained by the condensation of isothiosemicarbazide with the desired aldehyde under the same conditions. It was obtained as a hydrobromide salt. To get the isothiosemicarbazone, the precipitate of hydrobromide salt was worked up with a dichloromethane-water mixture. The cyclized sulfanyl 1,2,4-triazole derivatives (CL1–CL3) were obtained by the intramolecular cyclization of hydrobromide salts of isothiosemicarbazones in methanol under reflux condition. The compound TL1 was already reported by Yamazaki *et al.* in 1975.^38^

### 3.2. Spectroscopic characterization of isothiosemicarbazones and their cyclized sulfanyl 1,2,4-triazole derivatives

The synthesized isothiosemicarbazones and their cyclized sulfanyl 1,2,4-triazole derivatives were well characterized by various spectroscopic techniques such as UV-vis, FT-IR, ^1^H NMR and ^13^C NMR, and HRMS.

#### 3.2.1. FT-IR and UV-vis spectroscopy

FT-IR spectra of the isothiosemicarbazones had a characteristic N–N stretching frequency in the range 1161–1168 cm^−1^.^37^ Absorption bands in the range 714–765 cm^−1^ corresponded to the C^2^–S^1^ bond, confirming the S-substitution.^37^ N–H stretching frequency was observed in the range 3434–3373 cm^−1^.^38^ Isothiosemicarbazones exhibited two types of C=N stretching frequencies in the range 1592–1606 and 1509–1570 cm^−1^ which corresponded to azomethine C^1^=N^1^ and C^2^=N^2^ groups, respectively.^46^ In addition to the above, TL2 displayed peaks at 1245, 1029 and 2926 cm^−1^ which could be assigned to asymmetric C–O–C, symmetric C–O–C and aliphatic C–H stretching vibrations of the OCH_3_ group, respectively. Similarly, TL3 exhibited additional peaks, apart from the characteristic bands of isothiosemicarbazones, at 1497 and 1328 cm^−1^, which corresponded to the asymmetric and symmetric stretching vibrations of the NO_2_ group, respectively.^47^ Upon cyclization of isothiosemicarbazones, there was a reduction in the C=N stretching frequency, and it appeared in the range 1467–1468 cm^−1^, and the N–N bond stretching frequency shifted to 1139–1175 cm^−1^. Absorption bands observed in the range 700–775 cm^−1^ were attributed to the C^2^–S^1^ stretching vibrations in the cyclized sulfanyl 1,2,4-triazole derivatives.^47^ Absence of N–H stretching frequency confirmed the cyclization of isothiosemicarbazones. Additional peaks at 1247, 1033 and 2922 cm^−1^ in the FT-IR spectrum of CL2 corresponded to asymmetric C–O–C, symmetric C–O–C and aliphatic C–H stretching vibrations of the OCH_3_ group, respectively. The cyclized sulfanyl 1,2,4-triazole compound CL3 exhibited peaks at 1497 and 1328 cm^−1^ which were ascertained to the asymmetric and symmetric vibrations of NO_2_, respectively (Fig. S1, S6, S11, S16, S21 and S26).

UV-vis spectra of the isothiosemicarbazones (TL1–TL3) and their cyclized sulfanyl 1,2,4-triazole derivatives CL1–CL3 were recorded in acetonitrile. It was observed that the electronic spectra of the isothiosemicarbazones showed strong absorption bands in the range 215–232 nm which corresponded to π → π* transitions of the aromatic rings, and absorption bands in the range 318–382 nm represented n → π* transitions of the conjugated azomethine groups.^35,46,48^ The absorption maxima displayed in the UV-vis spectra of cyclized sulfanyl 1,2,4-triazole derivatives in the range 222–258 nm could be assigned to π → π* transitions of the 1,2,4-triazole ring.^49^ Nitro-substituted isothiosemicarbazone (TL3) and its cyclized sulfanyl 1,2,4-triazole derivative (CL3) exhibited an additional absorption band at 250 and 308 nm, respectively, which corresponded to the n → π* transition of the nitro group (Fig. S2, S7, S12, S17, S22 and S27).

#### 3.2.2. ^1^H and ^13^C NMR spectroscopy

All the NMR spectra were recorded in DMSO-*d*_6_ or CDCl_3_ solvent. For DMSO-*d*_6_, residual and moisture peaks were observed at 2.51 and 3.33 ppm, respectively and for CDCl_3_, they were at 7.26 and 1.56 ppm, respectively. In the ^1^H NMR spectra of isothiosemicarbazones, the deshielded singlet at 8.33–8.16 ppm was assigned to the imine proton.^46,50^ The benzylic CH_2_ and NH protons were found to resonate in the regions 4.21–4.17 and 7.04–6.56 ppm, respectively.^38^ The signals in the region 2.91–2.84 ppm represented the terminal *N*-methyl protons. In addition, the methoxy group present in TL2 was observed at 3.77 ppm.^50^ In the ^13^C NMR spectra, C=N carbons (C^1^ and C^2^) were observed in the most deshielded region (165.26–148.35 ppm). Aliphatic carbons of the terminal *N*-methyl and benzylic CH_2_ groups were found to resonate in the shielded regions 30.75–30.88 and 33.85–33.89 ppm, respectively. Further, the methoxy carbon of TL2 was observed at 55.67 ppm (Fig. S3, S4, S8, S9, S13 and S14).^50^ As the compound exists as an *E*/*Z* isomeric mixture in the solution state, all major peaks were accompanied by minor ones.^35,38^ The integrations corresponding to the minor isomer were excluded to ensure better interpretation of the spectra. The *E*/*Z* isomerism of isothiosemicarbazones is discussed in detail in Section 3.4.

In the ^1^H NMR spectra of cyclized sulfanyl 1,2,4-triazole derivatives, the disappearance of the signals corresponding to imine and NH protons confirmed the cyclization of isothiosemicarbazones. Upon ring closure, the benzylic CH_2_ and *N*-methyl protons were deshielded (4.32–4.43 and 3.25–3.53 ppm, respectively). In the ^13^C NMR spectra, the C=N carbons (C^1^ and C^2^) were observed to be shifted to 150.91–161.14 ppm. There is no significant shift in the chemical shift value of the methoxy carbon in the spectrum of CL2 on cyclization. Aliphatic carbons of the *N*-methyl (31.49–32.36 ppm) and benzylic CH_2_ (37.58–38.98 ppm) groups were found to be deshielded when compared to those of the corresponding isothiosemicarbazones (Fig. S18, S19, S23, S24, S28 and S29).

#### 3.2.3. Mass spectrometry

Base peaks for the isothiosemicarbazones (TL1–TL3) and their cyclized sulfanyl 1,2,4-triazole derivatives (CL1–CL3) in their HRMS were observed at 284.1229, 314.1326, 329.1072, 282.1067, 312.1172 and 327.0918, respectively, corresponding to [M + H]^+^, which were in good agreement with the calculated values. Therefore, the formation of the compounds was confirmed (Fig. S5, S10, S15, S20, S25 and S30).

### 3.3. Crystallographic characterization of isothiosemicarbazones and their cyclized sulfanyl 1,2,4-triazole derivatives

**Fig. 4:**
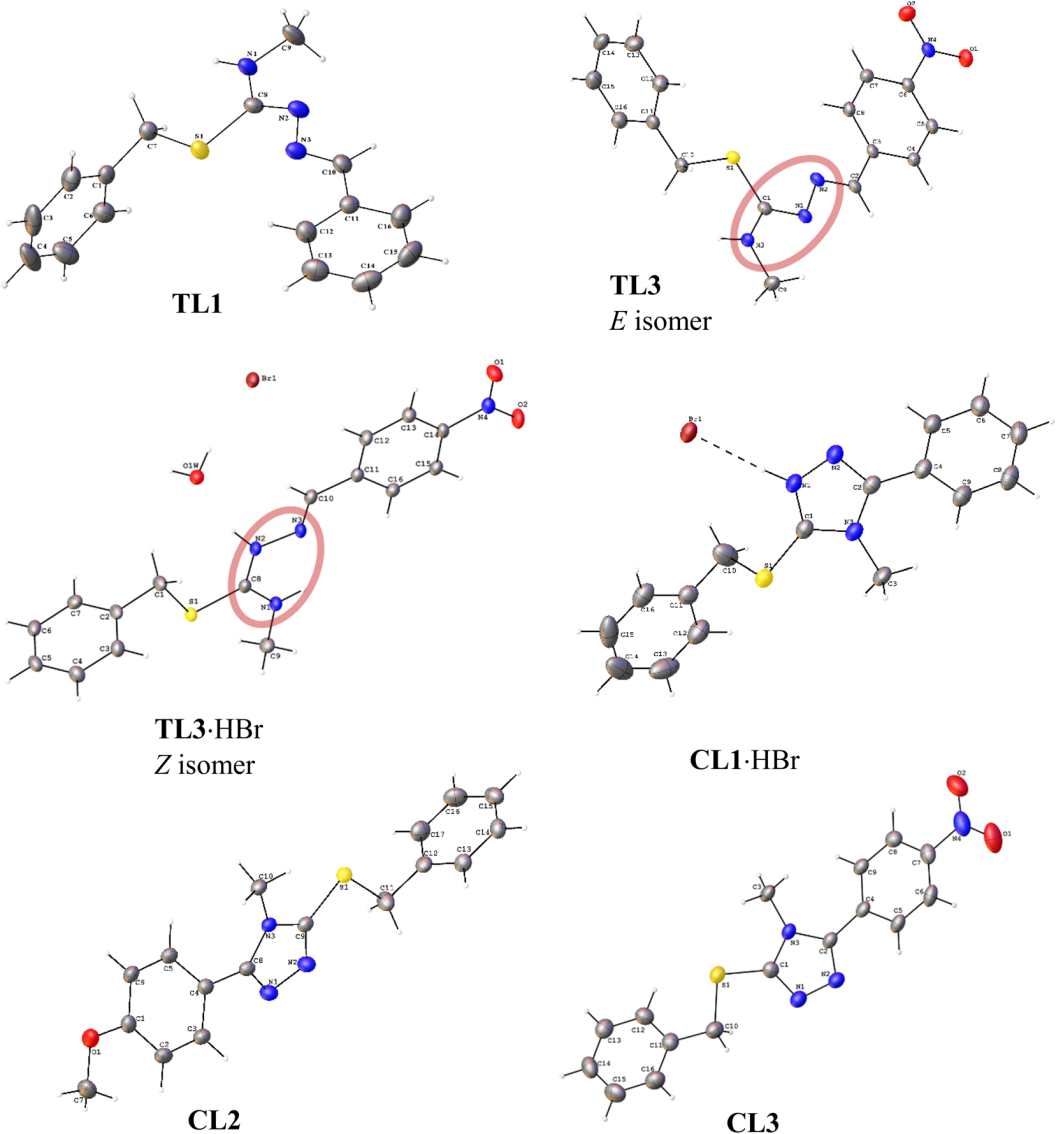
Molecular structures of isothiosemicarbazones [TL1, TL3 (*E* isomer) and TL3·HBr (*Z* isomer)] and cyclized sulfanyl 1,2,4-triazole derivatives (CL1·HBr, CL2 and CL3).

The molecular structures derived from the single crystal XRD analyses are shown in Fig. 4, which are consistent with the structures proposed from spectroscopic data for the isothiosemicarbazones and their cyclized sulfanyl 1,2,4-triazole derivatives. A summary of crystallographic data and refinement parameters is given in Tables S1 and S2. TL1 was obtained as yellow block-shaped crystals on recrystallization, and it was crystallized as a monoclinic crystal system with a space group of *P*2_1_/*c*. The *E* isomer of TL3 was crystallized as orange needle-shaped crystals, and the *Z* isomer of TL3·HBr as yellow block-shaped crystals. TL3 and TL3·HBr were crystallized in a monoclinic and triclinic fashion with a space group of *P*2_1_ and *P*-1, respectively. For TL1, C=N bond length was found to be 1.2777 (C^1^=N^1^) and 1.2997 (C^2^=N^2^) Å, comparable to the standard C=N length (1.27 Å). N^1^–N^2^ and C^2^–S^1^ had a single bond character with the bond lengths of 1.4043 and 1.7699 Å, respectively, in accordance with the standard bond length values of N–N (1.447 Å) and C–S (1.82 Å). In TL3 and TL3·HBr, C=N bond length was observed as 1.281 and 1.278 Å (C^1^=N^1^), and 1.312 and 1.336 Å (C^2^=N^2^), respectively. The small increment in the C^2^=N^2^ bond length in the hydrobromide salt was due to the interaction of H^+^ with the N^2^ atom. There is no significant difference in the N^1^–N^2^ bond length of TL3 and TL3·HBr, which were 1.387 and 1.3907 Å, respectively. However, a slight shortening of the C^2^–S^1^ bond was noted in TL3·HBr (1.7411 Å) compared to TL3 (1.7681 Å).

The cyclized sulfanyl 1,2,4-triazole compounds crystallized in a monoclinic system with a space group *P*2_1_/*c* (CL1·HBr and CL3) or *C*2/*c* (CL2). CL1·HBr and CL2 formed colourless block-shaped crystals, whereas CL3 yielded yellow block-shaped crystals. CL1 was recrystallized with HBr, in which H^+^ was interacting with N^2^. Upon cyclization of isothiosemicarbazones, the C=N bond length got increased from 1.277–1.281 to 1.307–1.319 Å, which implied the partial double bond character of C=N as a consequence of electron delocalisation within the triazole ring. A new C^1^–N^3^ bond was formed during the ring closure, whose length was observed in the range 1.368–1.374 Å.

### 3.4. *E*/*Z* isomerization of isothiosemicarbazones

Isothiosemicarbazones exist in two isomeric forms (*E* and *Z*) concerning the C^2^=N^2^ bond as shown in Fig. 4. The *Z* isomer of TL3 was recrystallized as its hydrobromide salt (yellow block-shaped), whereas the corresponding *E* isomer was recrystallized as orange needle-shaped crystal from its saturated acetonitrile solution with a few drops of methanol. The crystals were mechanically separated and analyzed by single crystal XRD. ^1^H NMR spectrum was recorded for the *E* isomer, which was same as that of synthesized TL3 consisting of both the isomers, suggesting the isomerization in the solution state.

**Fig. 5:**
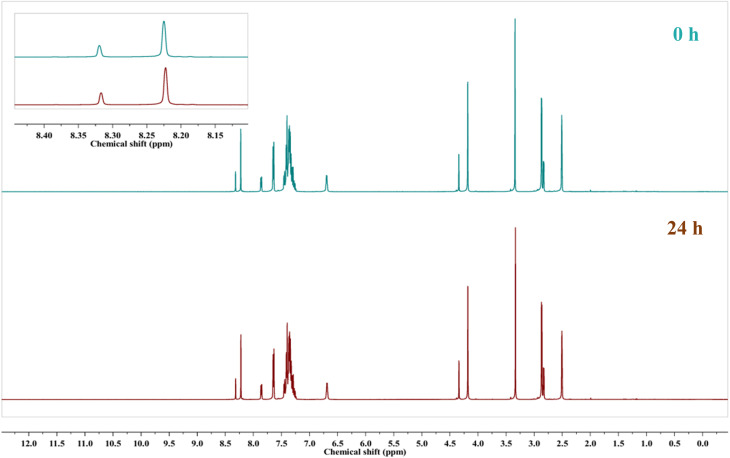
^1^H NMR spectra of TL1 in DMSO-*d*_6_ for a period of 24 h.

**Fig. 6:**
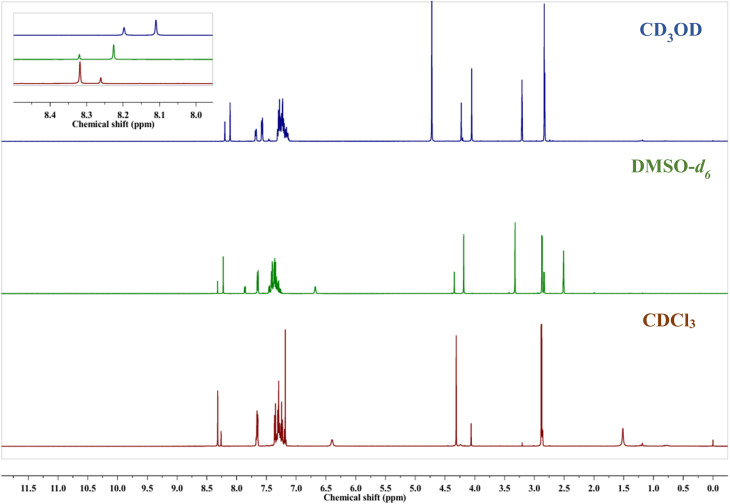
^1^H NMR spectra of TL1 in CD_3_OD, DMSO-*d*_6_ and CDCl_3_.

To study the *E*/*Z* isomerization in the solution state,^38^ a time-dependent ^1^H NMR analysis of TL1 was performed in CDCl_3_ for a period of 24 h. Immediately following the sample preparation, ^1^H NMR spectrum was acquired. The same sample was reanalyzed after 24 h under identical conditions. No change was observed between the spectra recorded at 0 and 24 h, indicating that the *E*/*Z* equilibrium was attained rapidly (Fig. 5). Additionally, the ^1^H NMR spectrum shown in Fig. S31 clearly demonstrates the coexistence of *E*/*Z* isomers, as evidenced by the duplicated signals and corresponding integration values of the characteristic protons, such as the imine proton, *N*-methyl proton and benzylic CH_2_ protons. To understand the ratio of *E*/*Z* isomers in different solvents,^38^^1^H NMR spectra of TL1 were recorded in CD_3_OD, DMSO-*d*_6_ and CDCl_3_. The ratio of *E*/*Z* isomers was calculated by considering the integration of imine protons of *E* and *Z* isomers in the NMR spectra. As the polarity of the solvent increases, its ability to interrupt intramolecular hydrogen bonding present in the *Z* isomer also increases. Therefore, it was clearly seen that the *E*/*Z* ratio changed significantly in different solvents. The *E*/*Z* ratio was found to be 21.25 : 78.74, 62.89 : 37.10, 76.33 : 23.66 in CDCl_3_, CD_3_OD and DMSO-*d*_6,_ respectively (Fig. 6).

### 3.5. Mechanism of cyclization

**Scheme 3:**
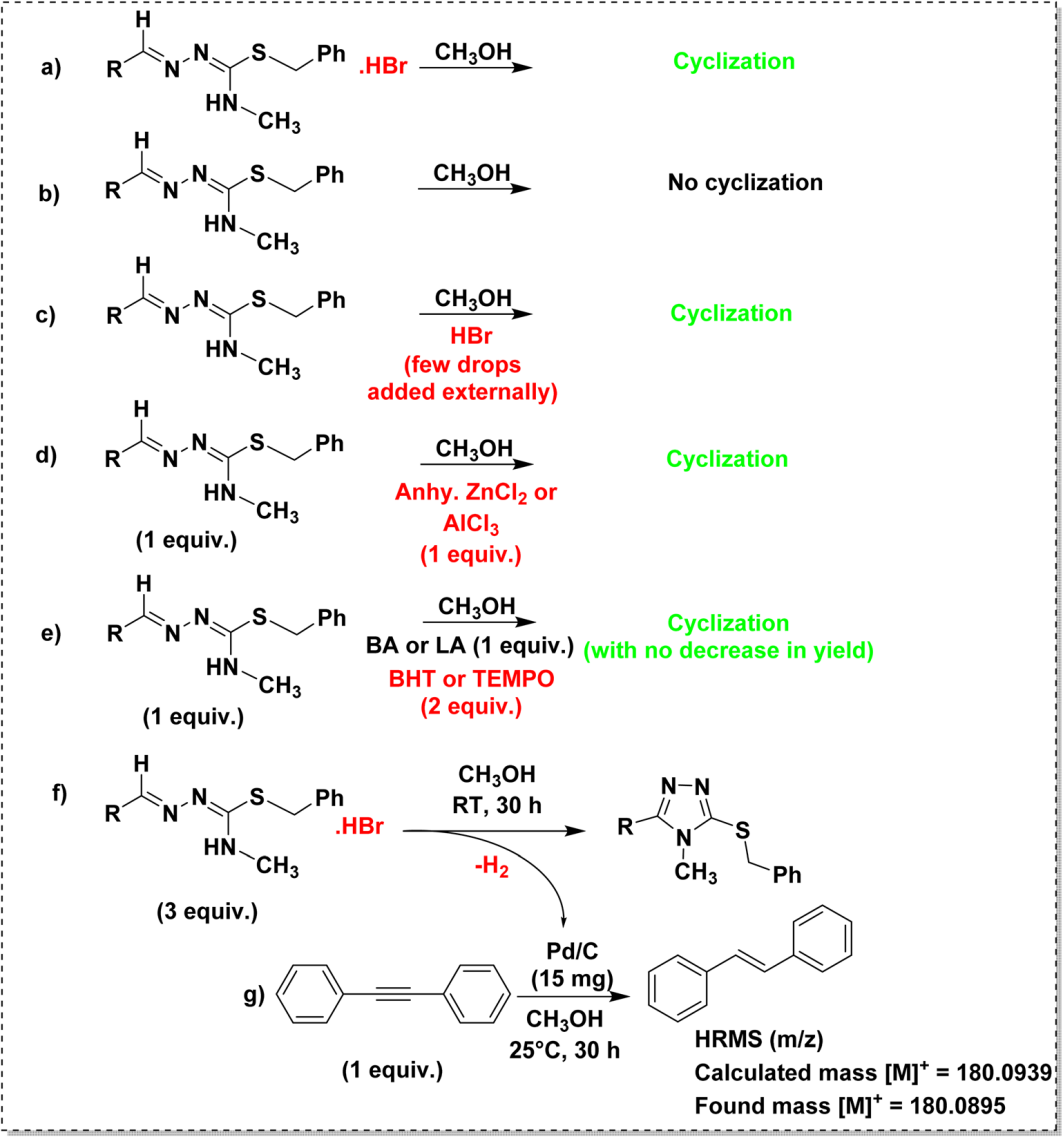
Controlled experiments for the intramolecular cyclization of isothiosemicarbazones.

**Scheme 4:**
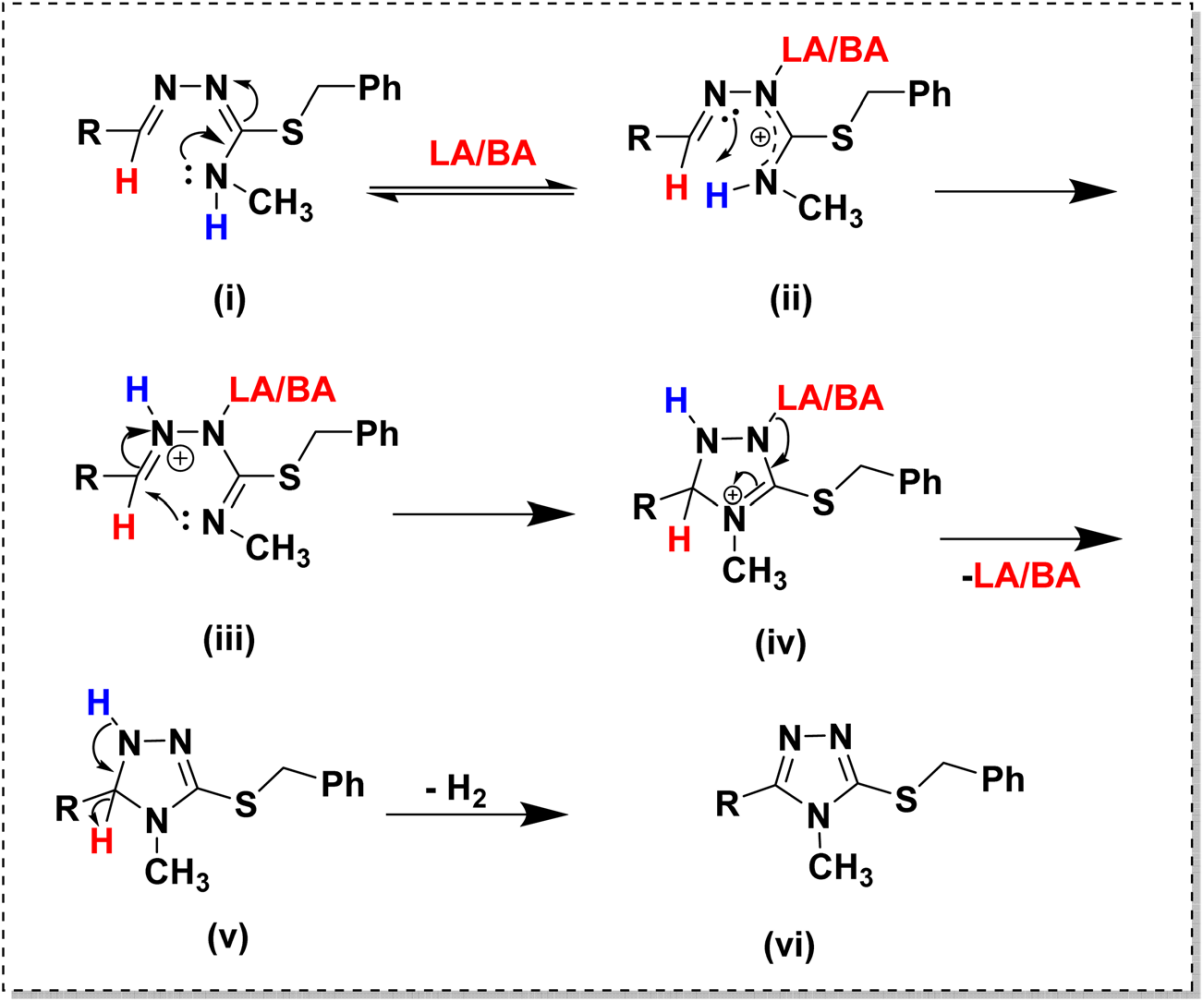
Plausible mechanistic pathway for the acid-catalyzed intramolecular cyclization of isothiosemicarbazone.

To understand the mechanism of cyclization, a few controlled experiments were performed as shown in Scheme 3. The hydrobromide salt of isothiosemicarbazone underwent cyclization in methanol under reflux condition (Scheme 3a). The same reaction was performed for the isothiosemicarbazone extracted by a dichloromethane-water workup (Scheme 3b), but no cyclization was observed. To get to know the role of HBr in cyclization, when a few drops of hydrobromic acid was added externally to the above reaction mixture (Scheme 3c), the cyclization occurred, which indicated that HBr acted as an acid catalyst for the reaction. The same reaction proceeded even in the presence of Lewis acid (LA), like anhydrous ZnCl_2_ or anhydrous AlCl_3_ in the place of Brønsted acid (BA), HBr (Scheme 3d). To explore the possible pathway of cyclization, reactions were performed in the presence of either a Brønsted acid or Lewis acid and radical scavenger like butylated hydroxytoluene (BHT) or 2,2,6,6-tetramethylpiperidin-1-oxyl (TEMPO) (Scheme 3e).^51^ The reactions proceeded with no decrease in yield, which implied that the cyclization was not proceeding *via* a free radical mechanism. So, it might have been followed an ionic mechanism. The crystal structure of TL3·HBr validated the first step [(ii) in Scheme 4] of the mechanism proposed for the acid-catalyzed cyclization of isothiosemicarbazones. Further, the intramolecular nucleophilic attack of the terminal nitrogen on the electrophilic azomethine carbon led to ring closure. The Brønsted/Lewis acid facilitates this process by activating the substrate toward intramolecular nucleophilic attack. In the following step, the LA/BA dissociates, resulting in the formation of a neutral intermediate [(v) in Scheme 4]. To achieve aromatic stabilization, the intermediate undergoes dehydrogenation with the loss of molecular hydrogen, thereby forming the aromatic triazole ring. To confirm the evolution of hydrogen during the cyclization of isothiosemicarbazones (Scheme 3d), a H-tube experiment^52^ was performed to trap the formed hydrogen, and used the same for the reduction of diphenylacetylene in the connected tube (Scheme 3g), as shown in Fig. S32. HRMS spectrum was recorded for the crude mixture in the right tube, and confirmed the presence of reduced product, further confirming the H_2_ evolution (Fig. S33).

### 3.6. Solution stability of sulfanyl derivatives of 1,2,4-triazoles

Solution stability studies for CL1–CL3 were performed in DMSO, DMSO-water (1 : 99 v/v), and PBS over a period of 24 h using UV-vis spectroscopy at room temperature. No new peaks or significant shifts in the absorption maxima were observed even after 24 h, indicating that the cyclized sulfanyl 1,2,4-triazole compounds were sufficiently stable to carry out their biological functions within the cell and are suitable for further *in vitro* studies (Fig. S34–S36).

### 3.7. Cytotoxic ability of the compounds

#### 3.7.1. *In vitro* cytotoxicity assessment *via* MTT assay

**Fig. 7:**
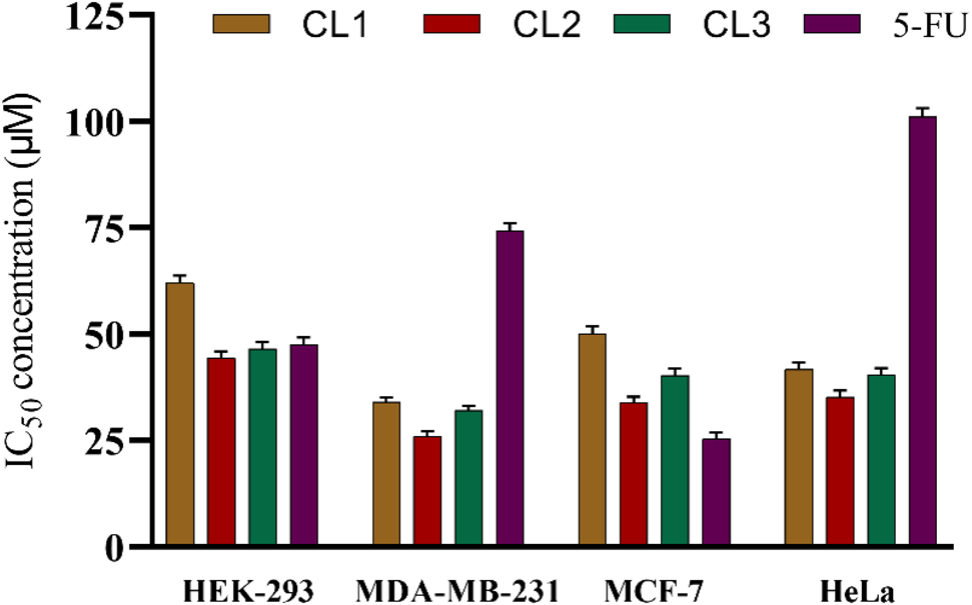
Comparison of IC_50_ values in various cell lines; data are presented as the mean IC_50_ (µM) ± SD.

The cytotoxic activity of compounds CL1–CL3 was evaluated using the MTT assay against MDA-MB-231, MCF-7 and HeLa cancer cell lines, with HEK-293 cells as a non-cancerous control and 5-FU as a reference drug. All the compounds exhibited cytotoxic effects across all the tested cancer cell lines. The compounds inhibited cancer cell growth with IC_50_ values that are mentioned in Table 1, surpassing the potency of 5-FU. Importantly, the compounds demonstrated high selectivity towards cancer cells, displaying substantially higher IC_50_ values in HEK-293 cells (Fig. 7). Among the cyclized sulfanyl 1,2,4-triazole compounds, the substituted derivatives CL2 (*p*-OCH_3_) and CL3 (*p*-NO_2_) showed enhanced activity across all the selected cell lines, indicating that substituent influences the biological activity; however, no definitive structure–activity relationship based on electronic effects of the substituent could be explained. Compared with 5-FU, CL2 showed higher cytotoxicity in MDA-MB-231 and HeLa cell lines, with IC_50_ values of 26.2 ± 1.4 and 35.2 ± 1.5, respectively. Methoxy substitution can enhance lipophilicity, thereby improving membrane permeability and facilitating favourable interactions with biological targets, which may contribute to enhanced anticancer activity.^43,53^ Conversely, the nitro-substituted derivative CL3 also exhibited good activity comparable to CL2. This observation may be attributed to the strong hydrogen bonding ability of the nitro group, which can promote effective interactions with the biological targets. In addition, nitro-containing compounds can function as prodrugs.^54^ Furthermore, the strong electron-withdrawing nature of the nitro group generates an electron deficient center within the molecule, favouring its interactions with biological targets possessing nucleophilic sites.^55^ Although electronic modulation has influence on biological activity, the present results did not reveal any direct relationship between cytotoxicity and the electron donating or electron withdrawing nature of the substituents.

**Table 1:** IC_50_ values of the cyclized sulfanyl 1,2,4-triazole derivatives and 5-FU against the tested cell lines after 24 h of incubation. The results are reported as the mean IC_50_ (µM) ± standard deviation (SD) of three independent experiments

Compound	HEK-293 (IC_50_ ± SD)	MDA-MB-231 (IC_50_ ± SD)	MCF-7 (IC_50_ ± SD)	HeLa (IC_50_ ± SD)
CL1	61.9 ± 1.8	34.3 ± 1.5	50.2 ± 1.7	41.7 ± 1.6
CL2	44.4 ± 1.6	26.2 ± 1.4	33.8 ± 1.5	35.2 ± 1.5
CL3	46.5 ± 1.7	32.3 ± 1.5	40.3 ± 1.6	40.4 ± 1.6
5-FU	47.5 ± 1.7	74.5 ± 1.9	25.4 ± 1.4	101.2 ± 2.0

#### 3.7.2. Detection of morphological alterations by AO/EB and Hoechst 33342 staining

**Fig. 8:**
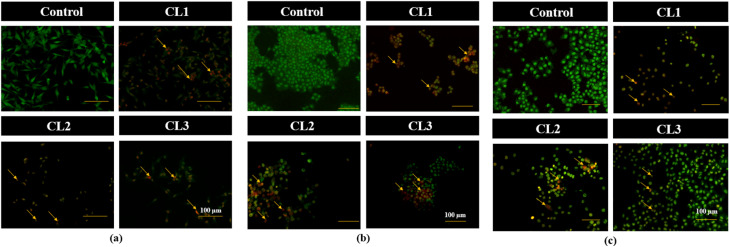
Dual AO/EB staining images (20× magnification) of untreated cell lines (control) and cyclized sulfanyl 1,2,4-triazole derivatives (CL1–CL3) treated MDA-MB-231 (a), MCF-7 (b) and HeLa (c) cancer cell lines.

**Fig. 9:**
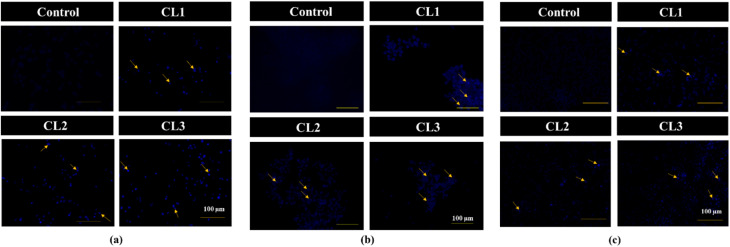
Hoechst 33342 staining images (20× magnification) of untreated cell lines (control) and cyclized sulfanyl 1,2,4-triazole derivatives (CL1–CL3) treated MDA-MB-231 (a), MCF-7 (b) and HeLa (c) cancer cell lines.

Cell staining assays, particularly dual AO/EB and Hoechst 33342, were employed to examine the morphological and nuclear alterations associated with drug-induced apoptosis in cancer cells. Given the pronounced cytotoxicity of the compounds against the tested cancer cell lines (MDA-MB-231, MCF-7 and HeLa), AO/EB (Fig. 8) and Hoechst 33342 (Fig. 9) staining methods were performed to confirm apoptotic cell death. AO permeates both healthy and damaged cells and emits green fluorescence, whereas EB selectively enters cells with compromised membrane integrity (damaged cells), producing orange to red fluorescence depending on the stage of apoptosis.^43,45^ The orange or red fluorescence emitted by the treated cells is a measure of the cells undergoing apoptosis. Following 24 h exposure to IC_50_ concentrations of compounds CL1–CL3, AO/EB-stained cells displayed green, orange and red fluorescence, indicating the presence of viable, early apoptotic and late apoptotic populations of cells, respectively. In Hoechst 33342 staining, the untreated cells displayed a uniform faint blue fluorescence, while drug-treated cells showed enhanced fluorescence intensity with characteristic apoptotic morphological changes.^43,45^ Increased fluorescence intensity is attributed to enhanced dye uptake and binding to condensed and fragmented nuclear DNA in apoptotic cells. The characteristic change in the morphology, could be attributed to the apoptotic changes (early and late), nuclear fragmentation, chromatin condensation and membrane blebbing on drug treatment. These observations confirmed the effective apoptosis induction by the compounds and validated the results obtained from the MTT assay.

#### 3.7.3. Assessment of mitochondrial membrane potential (MMP) by Rhodamine-123 staining

**Fig. 10:**
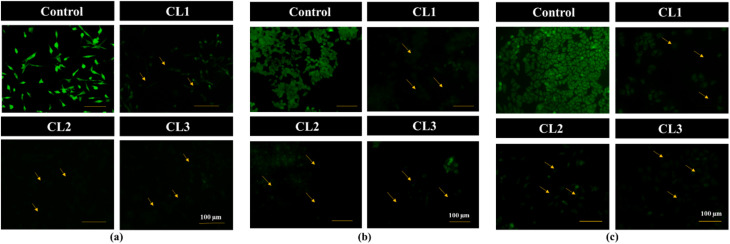
Rh-123 staining images (20× magnification) of untreated cell lines (control) and cyclized sulfanyl 1,2,4-triazole derivatives (CL1–CL3) treated MDA-MB-231 (a), MCF-7 (b) and HeLa (c) cancer cell lines.

Changes in MMP were investigated using Rhodamine-123 (Rh-123) dye, a cell-permeant cationic green fluorescent dye. Healthy cells exhibited strong Rh-123 fluorescence, reflecting intact mitochondrial membrane potential. Disruption of MMP can trigger apoptotic cell death through mitochondrial dysfunction.^45,56^ Following 24 h treatment of MCF-7, MDA-MB-231 and HeLa cancer cell lines with compounds CL1–CL3 (Fig. 10), a marked reduction in fluorescence intensity was observed for all the compounds compared with the control group, indicating significant depletion of MMP, and further confirming that apoptosis induced by the compounds proceeds predominantly through the mitochondrial pathway.

#### 3.7.4. Intracellular ROS generation

**Fig. 11:**
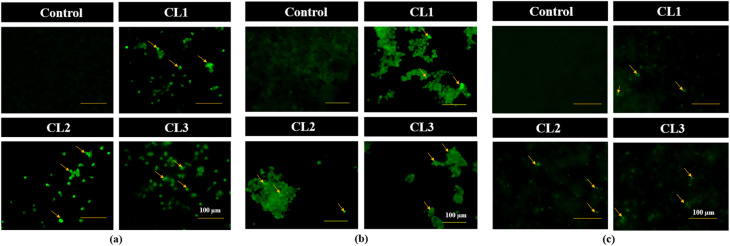
DCFH-DA staining images (20× magnification) of untreated cell lines (control) and cyclized sulfanyl 1,2,4-triazole derivatives (CL1–CL3) treated MDA-MB-231 (a), MCF-7 (b) and HeLa (c) cancer cell lines.

Maintenance of ROS level is crucial for regular cellular function, and disruption of this balance can induce cell apoptosis. DCFH-DA fluorescent staining assay is used to quantify the intracellular ROS levels. DCFH-DA is a non-polar dye that can easily diffuse into cells, where it is deacetylated by intracellular esterases to form non-fluorescent DCFH (2′,7′-dichlorodihydrofluorescein). This non-fluorescent compound is oxidized to fluorescent DCF (2′,7′-dichlorofluorescein) by intracellular ROS. The fluorescence intensity is directly proportional to the level of intracellularly generated ROS.^45,56^ ROS generation in MDA-MB-231, MCF-7 and HeLa cancer cells treated with CL1–CL3 was assessed using DCFH-DA (Fig. 11) to determine the involvement of oxidative stress in drug-induced cytotoxicity. Compared to untreated control cells, compound-treated cells showed elevated intracellular ROS levels.

## 4. Conclusions

In conclusion, a series of isothiosemicarbazones (TL1–TL3) and their corresponding sulfanyl-1,2,4-triazoles (CL1–CL3) were synthesized by varying the substituents from electron withdrawing to electron donating to assess their influence on cytotoxicity. The sulfanyl derivatives of 1,2,4-triazoles were obtained *via* an acid-catalyzed intramolecular cyclization, and all the compounds were comprehensively characterized by FT-IR, UV-vis, ^1^H NMR and ^13^C NMR spectroscopic and HRMS techniques, and further validated with single crystal XRD. *E*/*Z* isomerism exhibited by the isothiosemicarbazones was investigated through detailed NMR spectroscopic studies and single crystal XRD. Moreover, mechanistic aspects of the acid-catalyzed intramolecular cyclization were explored, and it was concluded that cyclization proceeded *via* an ionic mechanism with the evolution of hydrogen as a byproduct. Biological evaluation revealed that the cyclized sulfanyl 1,2,4-triazole derivatives exhibited significant cytotoxicity against MDA-MB-231, MCF-7 and HeLa cancer cell lines, with lower toxicity toward normal cell line HK-293, suggesting good selectivity of the compounds to the cancer cells. Substituted derivatives CL2 and CL3, bearing *p*-OCH_3_ and *p*-NO_2_, respectively, exhibited enhanced activity across all the tested cancer cell lines than the unsubstituted analogue. The present study suggests that electronic modulation influences cytotoxicity, and the lipophilicity and hydrogen bonding ability of the substituents may also play an important role in enhancing the biological activity of the drug candidates. The mechanism of cell death was investigated using dual AO/EB, Hoechst 33342, Rh-123 and DCFH-DA fluorescence staining assays. The studies suggested that apoptosis might be induced through intracellular ROS generation and depletion of MMP. Overall, these findings highlight sulfanyl 1,2,4-triazoles as promising anticancer scaffolds. Further studies focusing on detailed mechanistic investigations, expanded structure–activity relationship analysis, and *in vivo* evaluation are warranted to advance their potential therapeutic development. Isothiosemicarbazones are a relatively underexplored class of compounds, despite being rich in coordination sites. In future work, we plan to synthesize organometallic compounds using isothiosemicarbazones as ligand systems, considering the growing importance of organometallic compounds in anticancer drug development.

## Author contributions

Kallivalappil Snisha: conceptualization, methodology, investigation, data curation, formal analysis, writing – original draft, writing – review & editing. Mano Chitra Karthikeyan: methodology, investigation, data curation, formal analysis, writing – review & editing. Nattamai Bhuvanesh: data curation, formal analysis. Antony Joseph Velanganni Arockiam: data curation, formal analysis, writing – review & editing. Ramasamy Karvembu: supervision, conceptualization, writing – review & editing.

## Conflicts of interest

There are no conflicts of interest to declare.

## Supplementary Material

RA-016-D6RA00822D-s001

RA-016-D6RA00822D-s002

## Data Availability

CCDC 2506364 (TL1), 2506370 (TL3·HBr), 2506371 (TL3), 2506359 (CL1·HBr), 2506360 (CL2) and 2506361 (CL3) contain the supplementary crystallographic data for this paper.^57*a*–*f*^ Supplementary information (SI): experimental procedures and spectral data (FT-IR, UV-vis, ^1^H & ^13^C NMR and HRMS). See DOI: https://doi.org/10.1039/d6ra00822d.
