# Supplementary material for: Synthesis of sulfanyl derivatives of 1,2,4-triazoles via an acid catalyzed intramolecular cyclization of isothiosemicarbazones: structural characterization, E/Z isomerism, mechanistic insights and in vitro cytotoxicity
Source: RSC Adv. 2026 Mar 10;16(15):13433–46. doi: 10.1039/d6ra00822d (PMC12973506; doi:10.1039/d6ra00822d)
Supplement: RA-016-D6RA00822D-s001 [file RA-016-D6RA00822D-s001.pdf]

Supplementary Information (SI) for RSC Advances.
This journal is © The Royal Society of Chemistry 2026

Electronic Supplementary Information

for

Synthesis of sulfanyl derivatives of 1,2,4-triazoles *via* an acid catalyzed intramolecular cyclization of isothiosemicarbazones: Structural characterization, *E*/*Z* isomerism, mechanistic insights and *in vitro* cytotoxicity

Kallivalappil Snisha[1], Mano Chitra Karthikeyan[2], Nattamai Bhuvanesh[3], Antony Joseph Velanganni Arockiam[2] and Ramasamy Karvembu[1,*]

[1]*Department of Chemistry, National Institute of Technology, Tiruchirappalli 620 015, India*

[2]*Molecular Oncology Laboratory, Department of Biochemistry, School of Life Sciences, Bharathidasan University, Tiruchirappalli 620 024, India*

[3]*Department of Chemistry, Texas A & M University, College Station, TX 77842, USA*

*E-mail (Corresponding author): kar@nitt.edu

Contents

Figure S1 FT-IR spectrum of **TL1** S4
Figure S2 UV-Vis spectrum of **TL1** S4
Figure S3 ^{1}H NMR spectrum of **TL1** in DMSO-d_6 .... S5
Figure S4 ^{13}C NMR spectrum of **TL1** in DMSO-d_6 .... S5
Figure S5 HRMS spectrum of **TL1** S6
Figure S6 FT-IR spectrum of **TL2** S7
Figure S7 UV-Vis spectrum of **TL2** S7
Figure S8 ^{1}H NMR spectrum of **TL2** in DMSO-d_6 .... S8
Figure S9 ^{13}C NMR spectrum of **TL2** in DMSO-d_6 .... S8
Figure S10 HRMS spectrum of **TL2** S9
Figure S11 FT-IR spectrum of **TL3** S10
Figure S12 UV-Vis spectrum of **TL3** S10
Figure S13 ^{1}H NMR spectrum of **TL3** in DMSO-d_6 .... S11
Figure S14 ^{13}C NMR spectrum of **TL3** in DMSO-d_6 .... S11
Figure S15 HRMS spectrum of **TL3** S12
Figure S16 FT-IR spectrum of **CL1** S13
Figure S17 UV-Vis spectrum of **CL1** S13
Figure S18 ^{1}H NMR spectrum of **CL1** in $CDCl_3$ S14
Figure S19 ^{13}C NMR spectrum of **CL1** in $CDCl_3$ S14
Figure S20 HRMS spectrum of **CL1** S15
Figure S21 FT-IR spectrum of **CL2** S16
Figure S22 UV-Vis spectrum of **CL2** S16
Figure S23 ^{1}H NMR spectrum of **CL2** in DMSO-d_6 .... S17
Figure S24 ^{13}C NMR spectrum of **CL2** in DMSO-d_6 .... S17
Figure S25 HRMS spectrum of **CL2** S18
Figure S26 FT-IR spectrum of **CL3** S19
Figure S27 UV-Vis spectrum of **CL3** S19
Figure S28 ^{1}H NMR spectrum of **CL3** in DMSO-d_6 .... S20
Figure S29 ^{13}C NMR spectrum of **CL3** in DMSO-d_6 .... S20
Figure S30 HRMS spectrum of **CL3** S21
Table S1 Crystallographic data and structure refinement details of isothiosemicarbazones S22
Table S2 Crystallographic data and structure refinement details of cyclized sulfanyl 1,2,4-triazole derivatives S23

Figure S31 ^{1}H NMR spectrum of **TL1** (*E*/*Z* isomeric mixture) in DMSO-d_6……..……....S24

Figure S32 Experimental setup for the reduction of diphenylacetylene experiment............S25

Figure S33 HRMS spectrum for the reduction of diphenylacetylene..................................S25

Figure S34 UV-Vis spectra of cyclized sulfanyl 1,2,4-triazole derivatives **CL1**-**CL3** in DMSO over a period of 24 h...S26

Figure S35 UV-Vis spectra of cyclized sulfanyl 1,2,4-triazole derivatives **CL1**-**CL3** in DMSO-water (1:99 v/v) over a period of 24 h S27

Figure S36 UV-Vis spectra of cyclized sulfanyl 1,2,4-triazole derivatives **CL1**-**CL3** in PBS (pH=7.4) over a period of 24 h S28

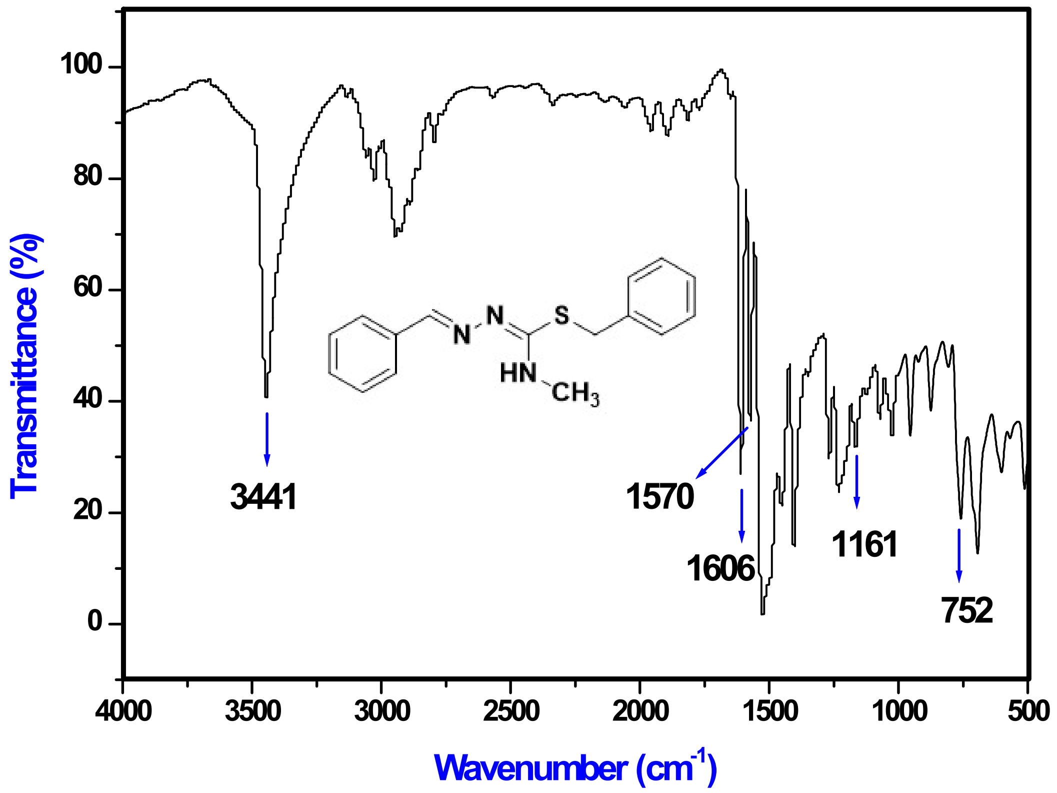

Figure S1 FT-IR spectrum of **TL1**

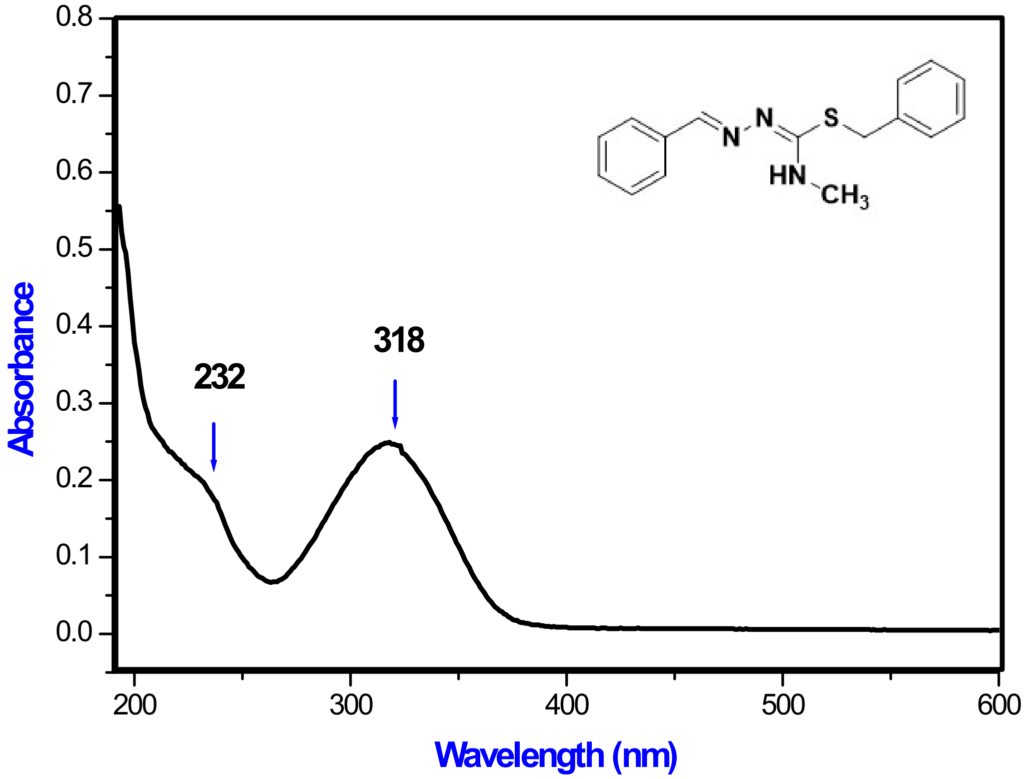

Figure S2 UV-Vis spectrum of **TL1**

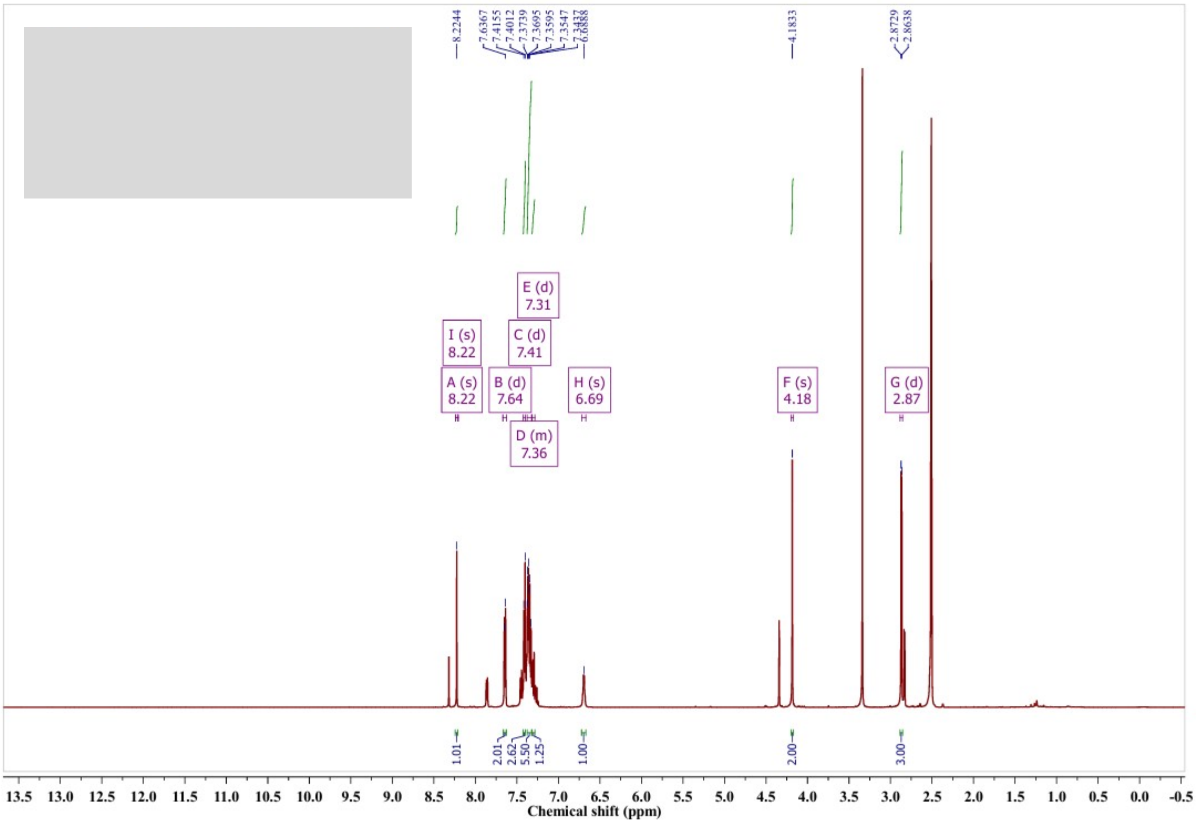

Figure S3 ^{1}H NMR spectrum of **TL1** in DMSO-d_6

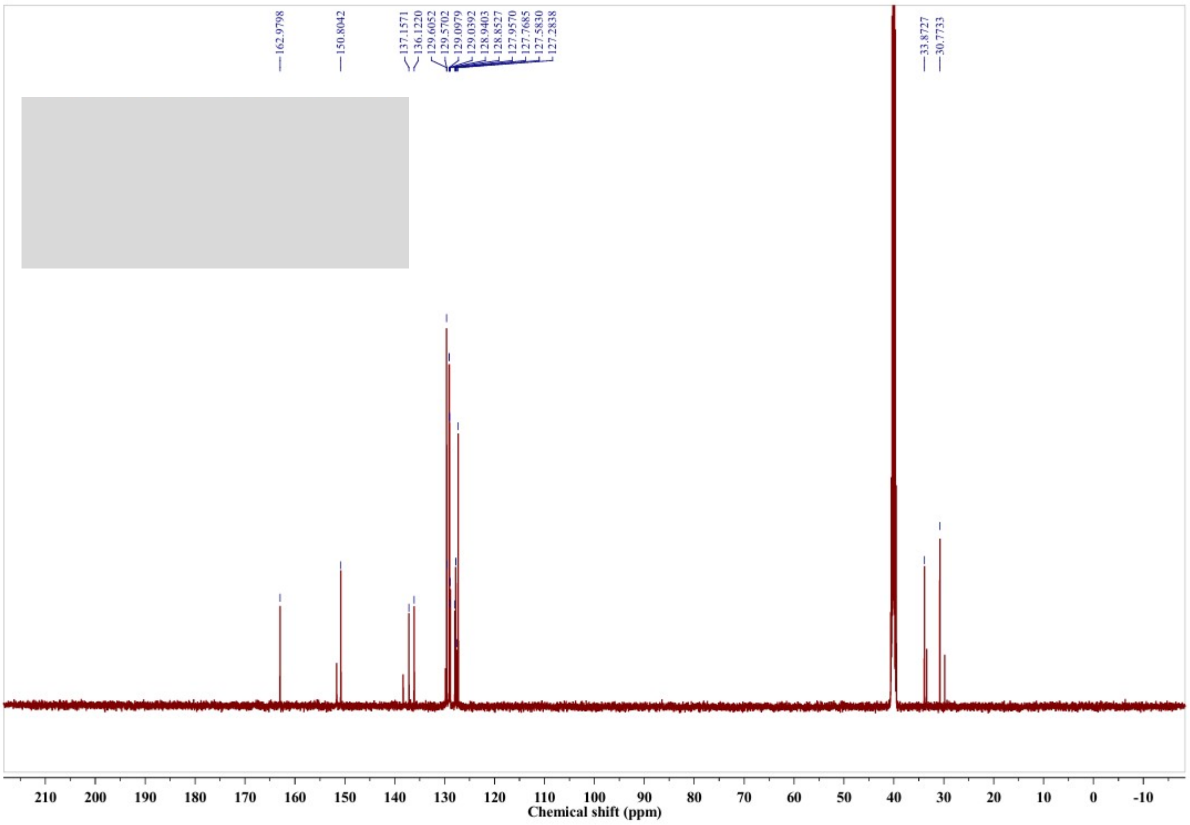

Figure S4 ^{13}C NMR spectrum of **TL1** in DMSO-d_6

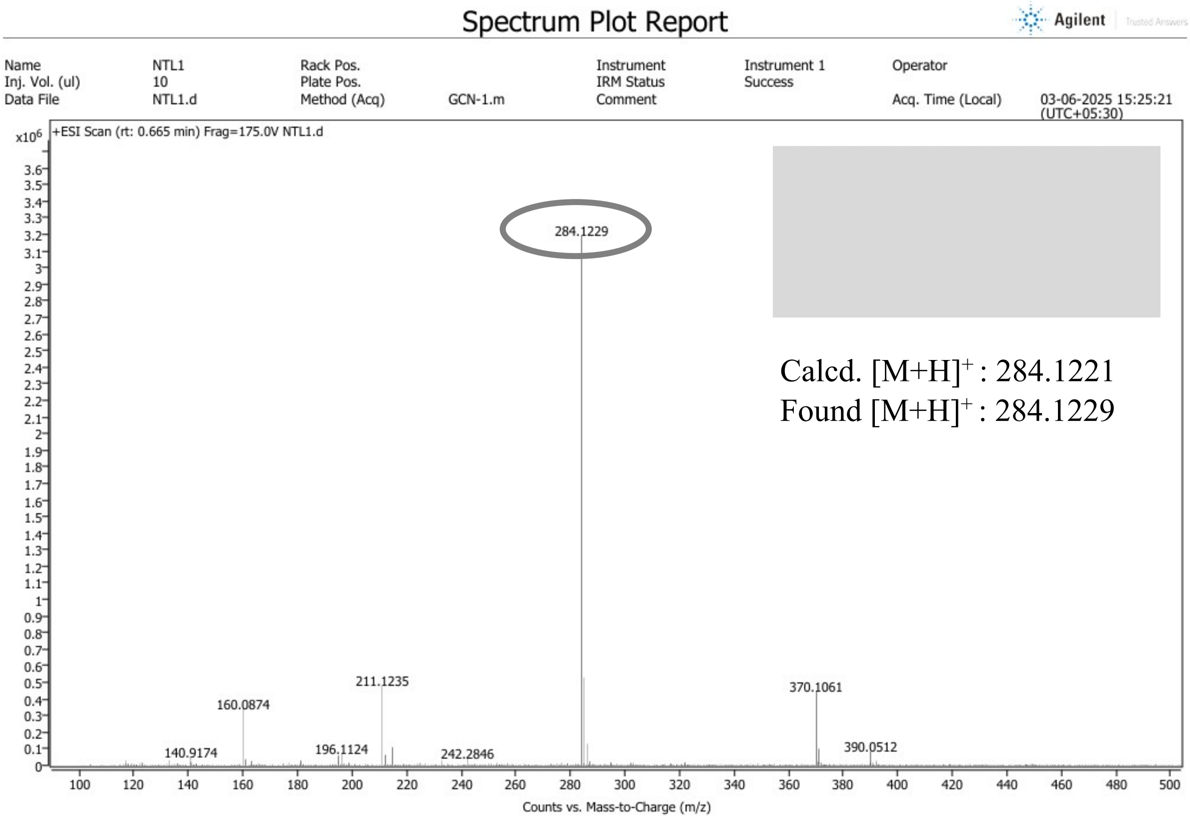

Figure S5 HRMS spectrum of **TL1**

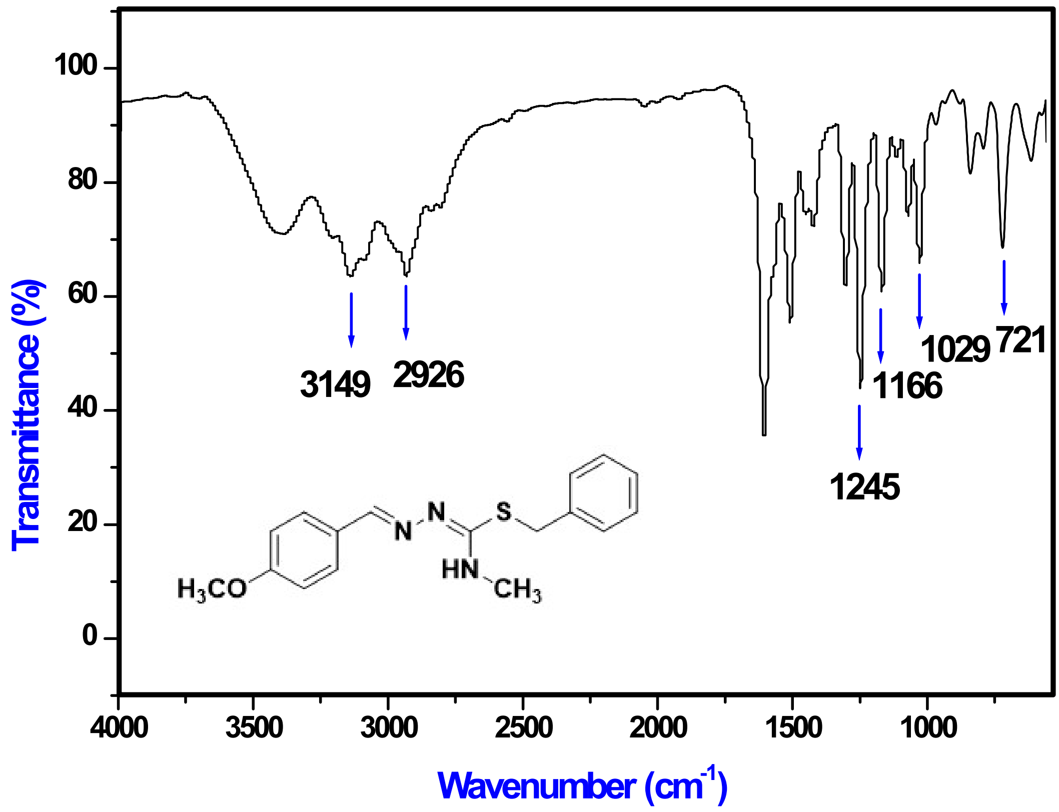

Figure S6 FT-IR spectrum of **TL2**

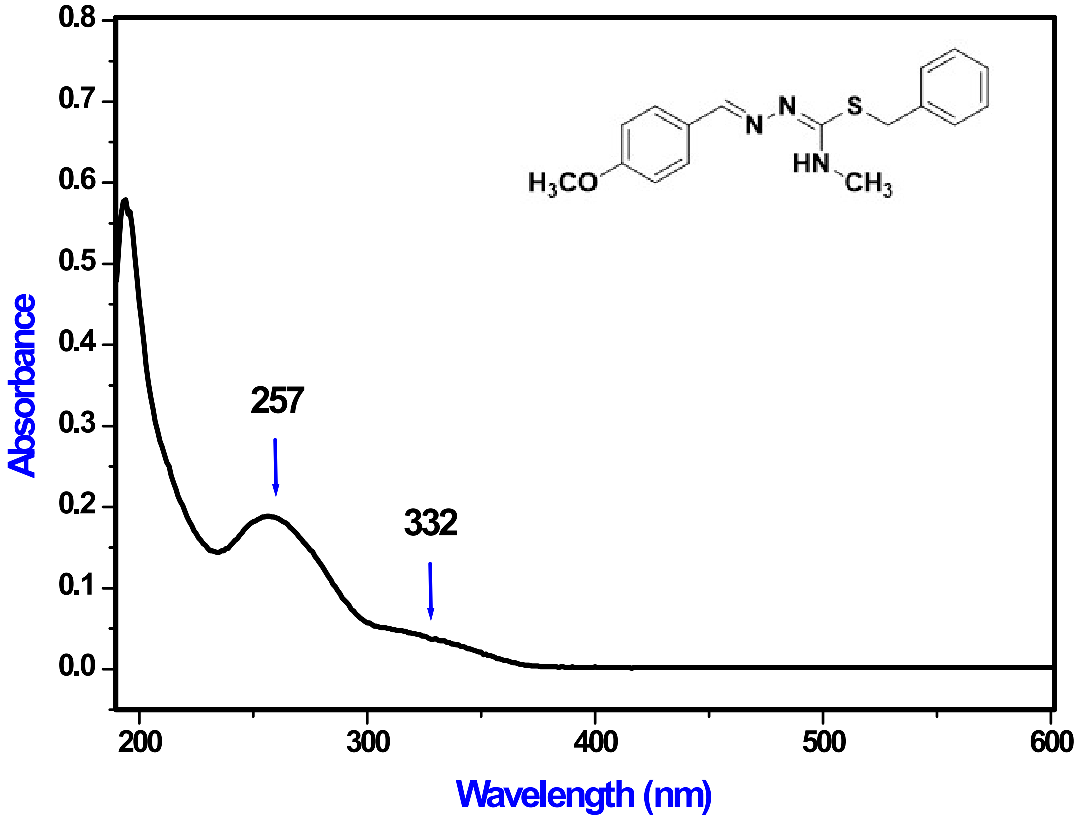

Figure S7 UV-Vis spectrum of **TL2**

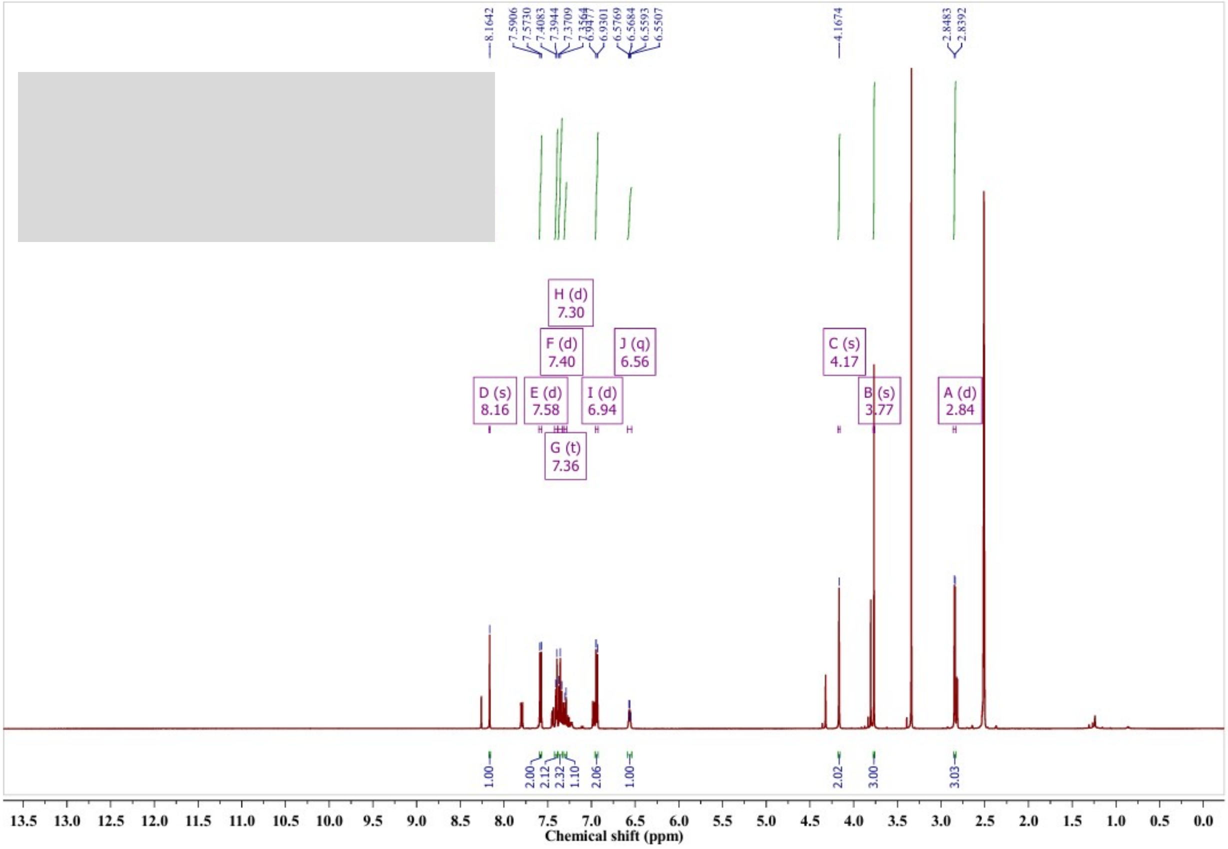

Figure S8 ^{1}H NMR spectrum of **TL2** in DMSO-d_6

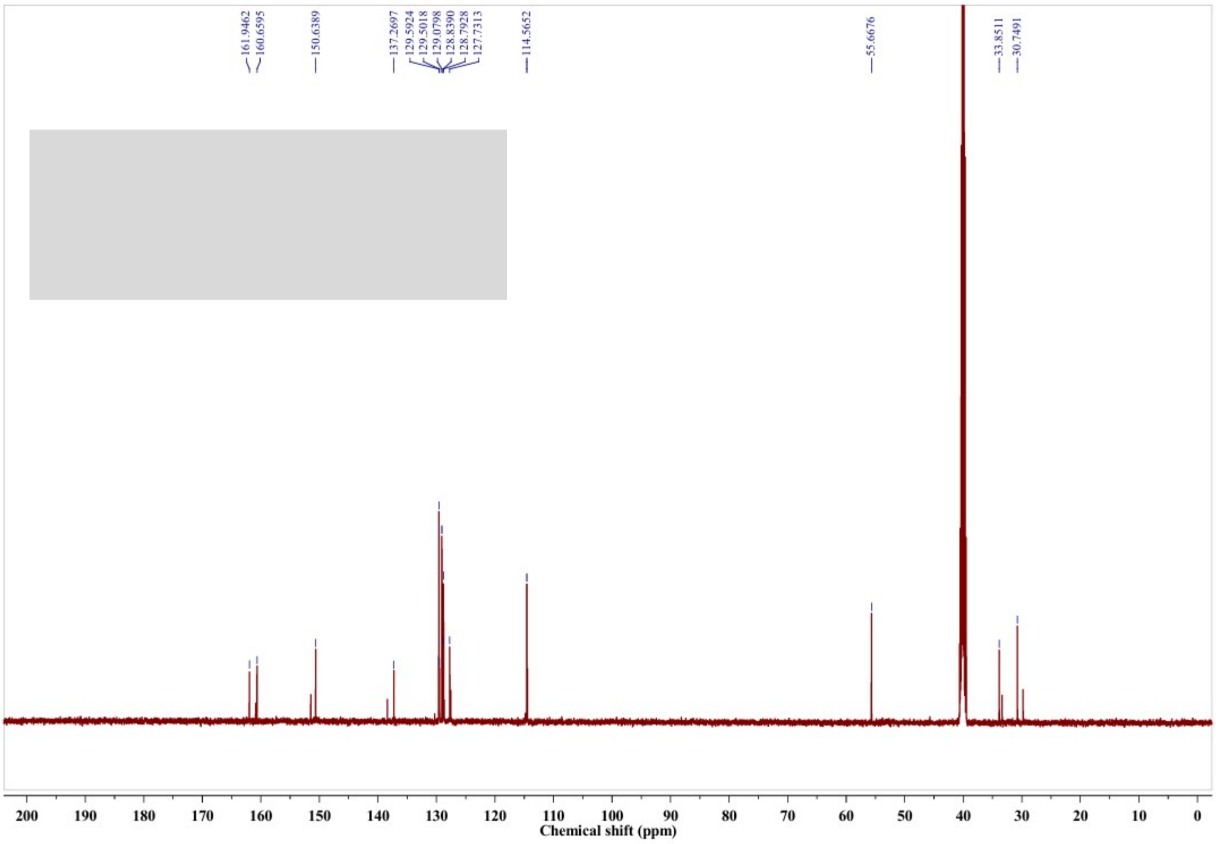

Figure S9 ^{13}C NMR spectrum of **TL2** in DMSO-d_6

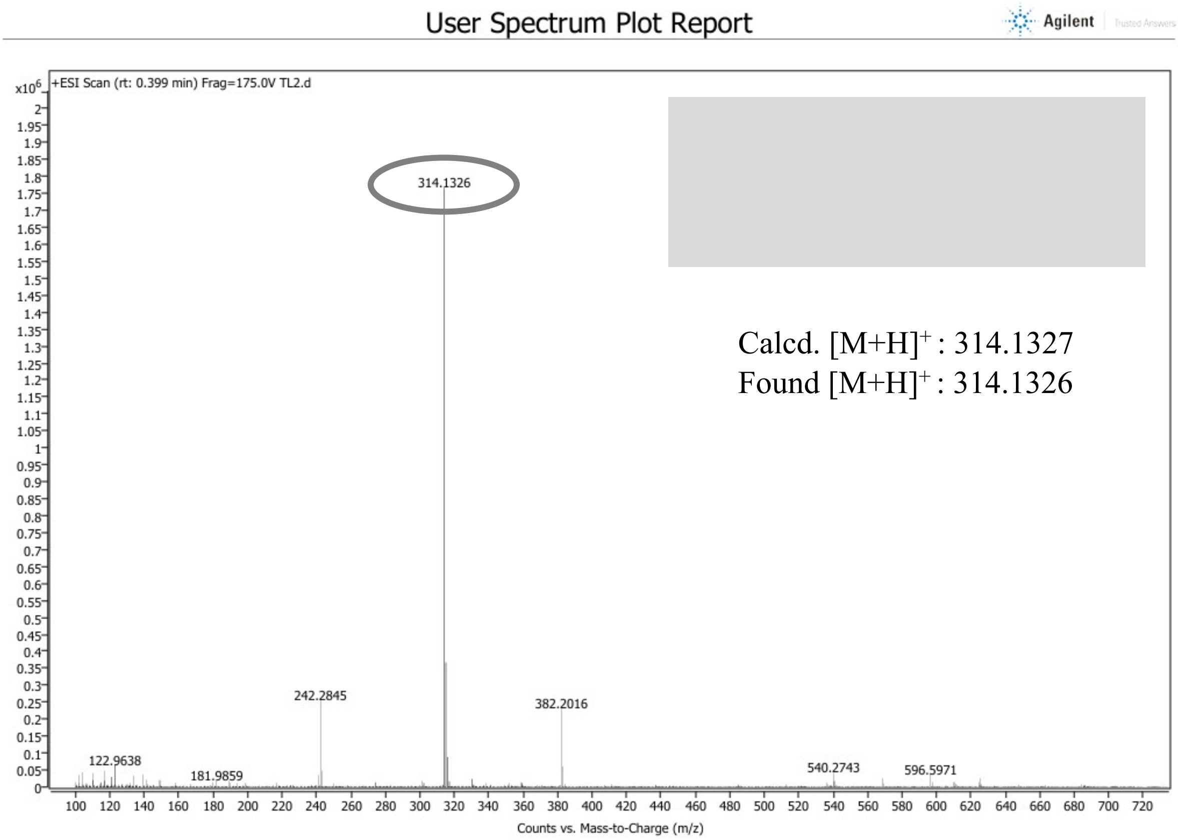

Figure S10 HRMS spectrum of **TL2**

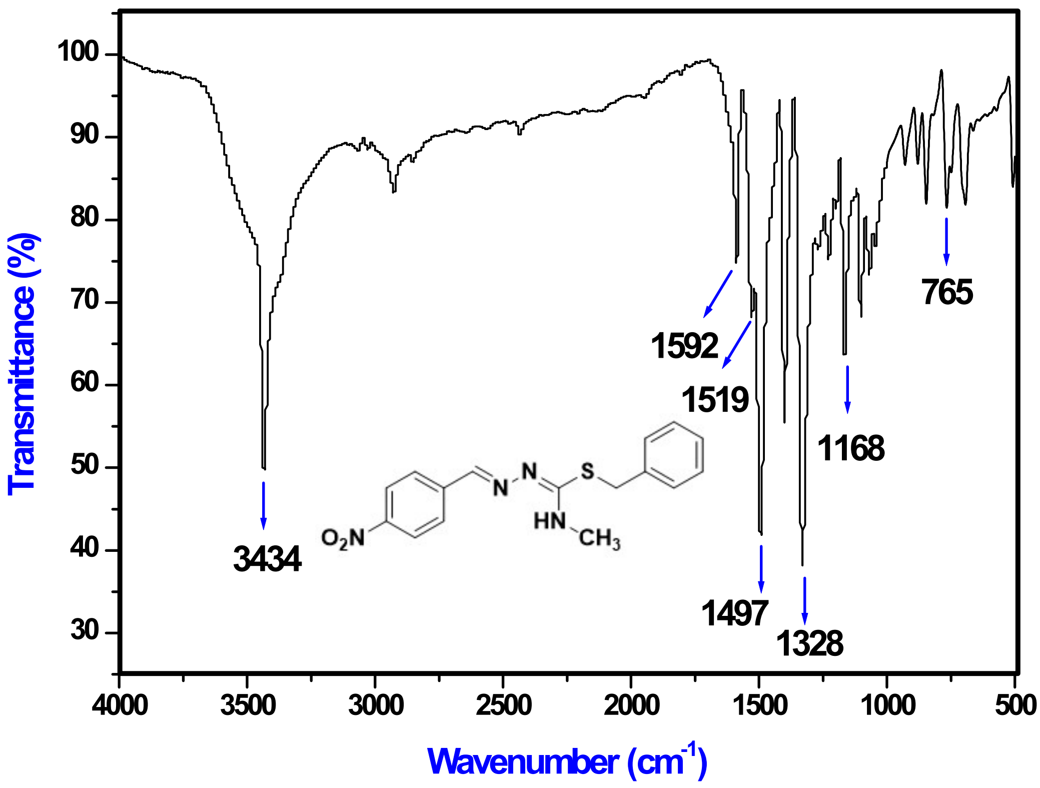

Figure S11 FT-IR spectrum of **TL3**

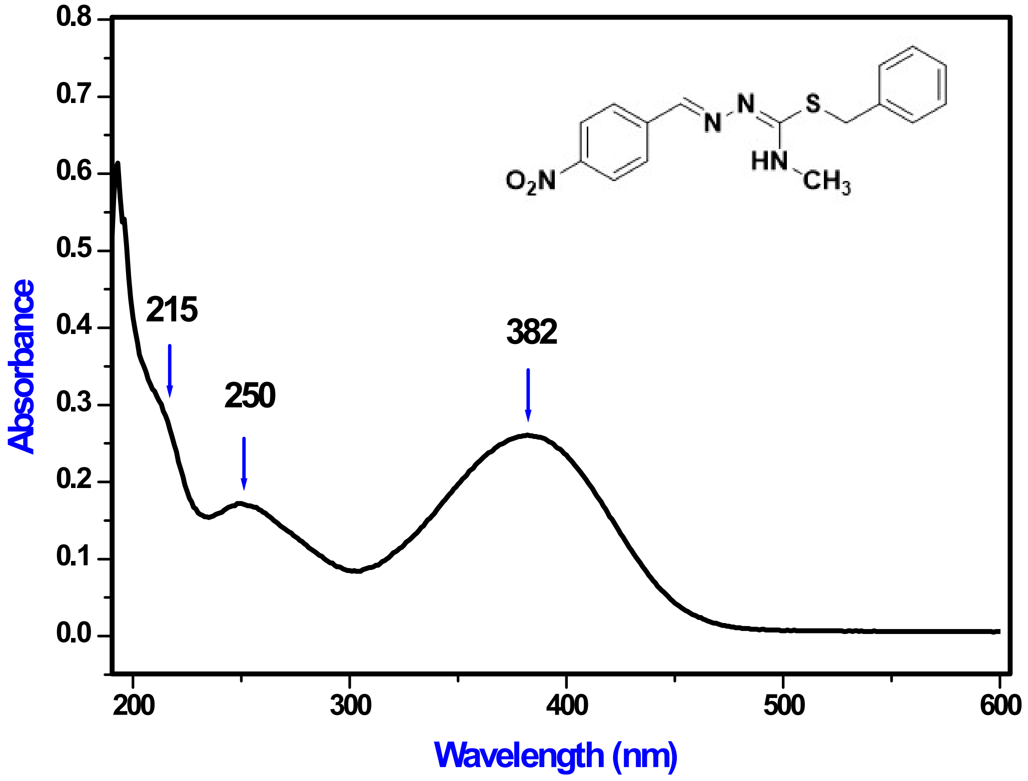

Figure S12 UV-Vis spectrum of **TL3**

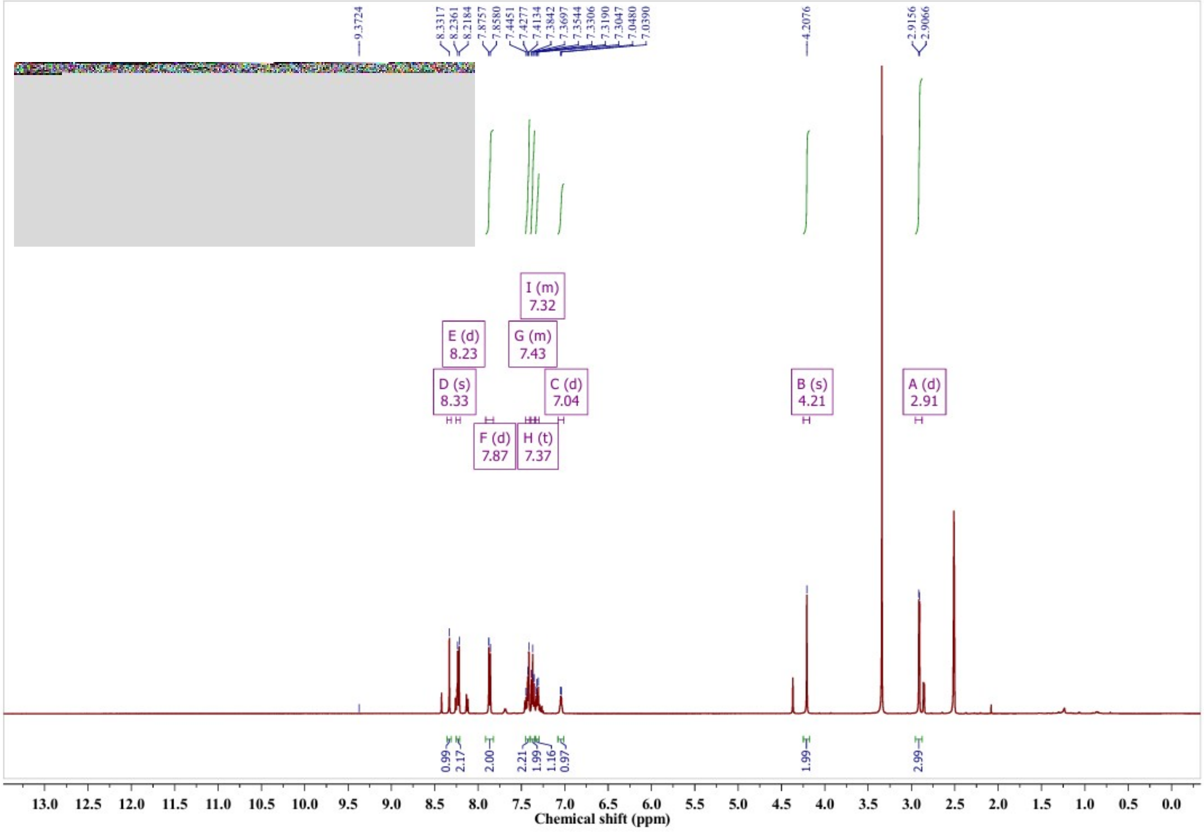

Figure S13 ^{1}H NMR spectrum of **TL3** in DMSO-d_6

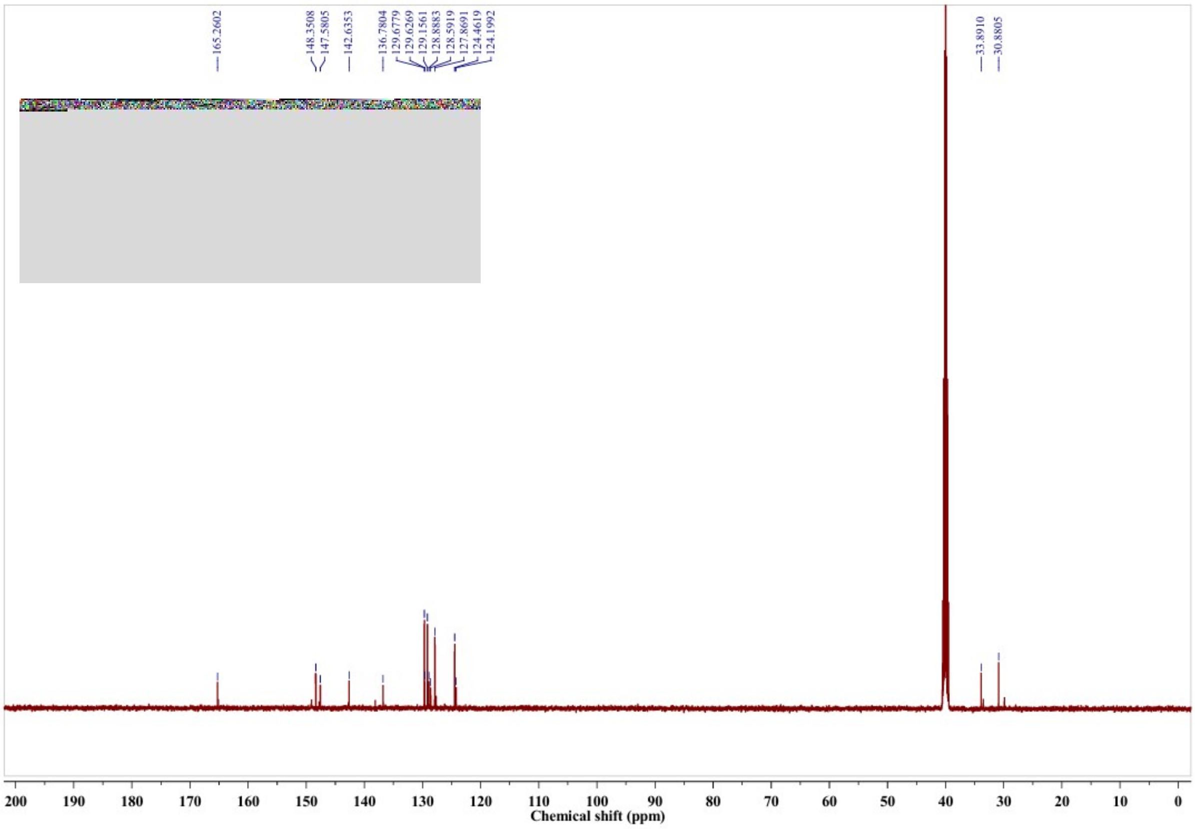

Figure S14 ^{13}C NMR spectrum of **TL3** in DMSO-d_6

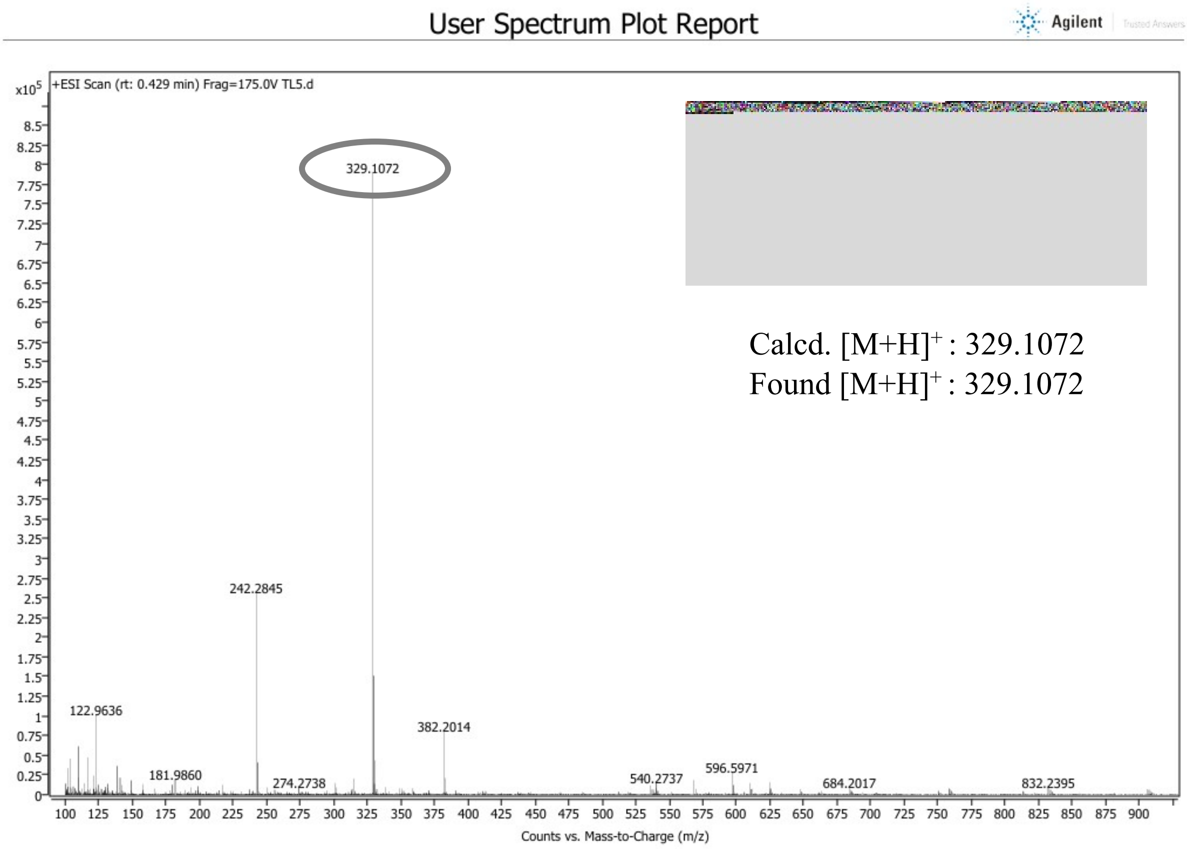

Figure S15 HRMS spectrum of **TL3**

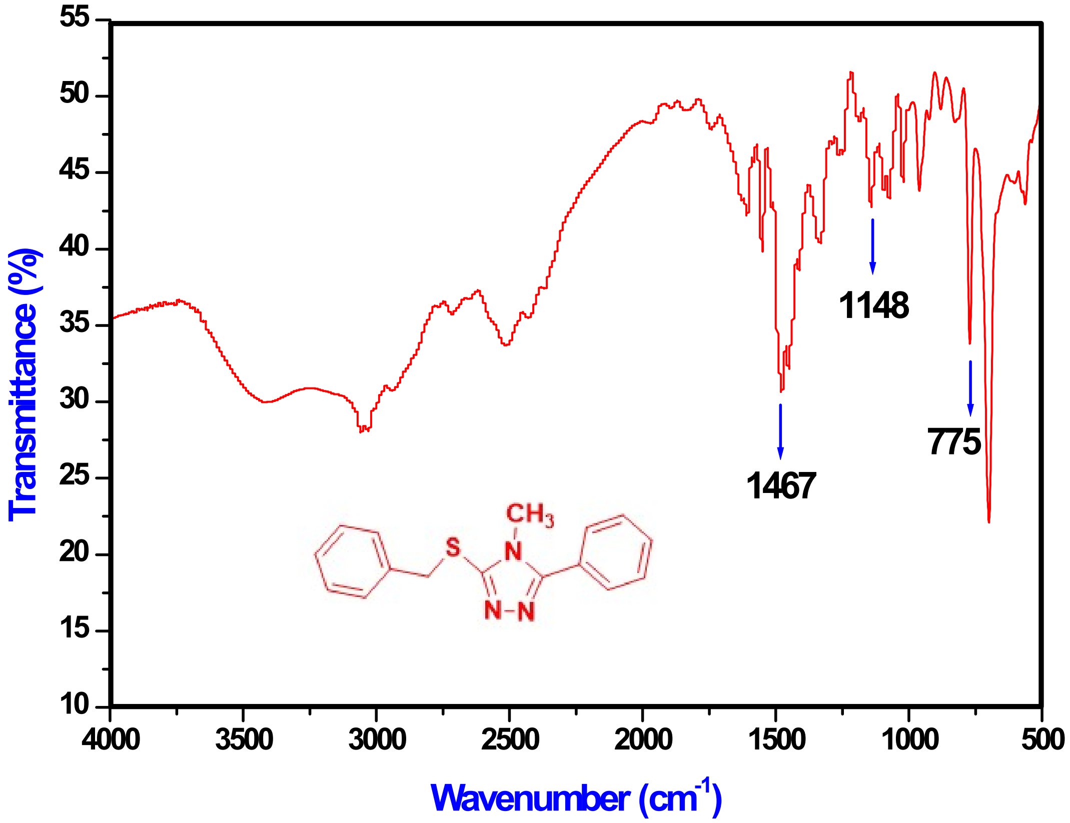

Figure S16 FT-IR spectrum of **CL1**

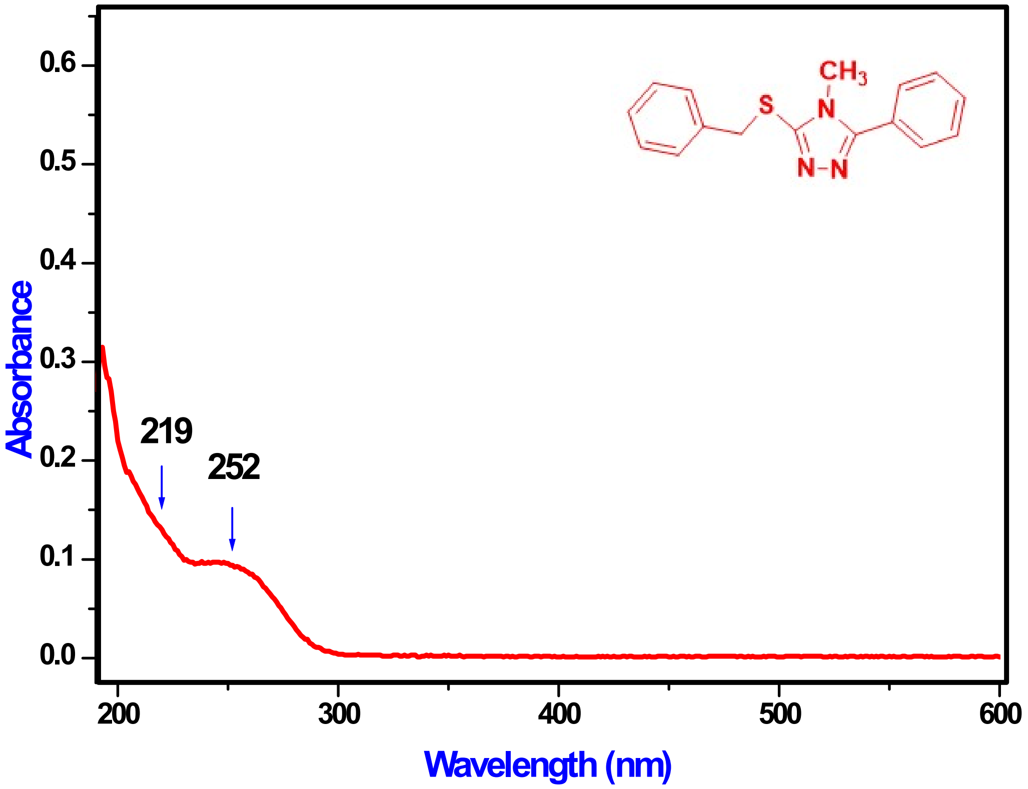

Figure S17 UV-Vis spectrum of **CL1**

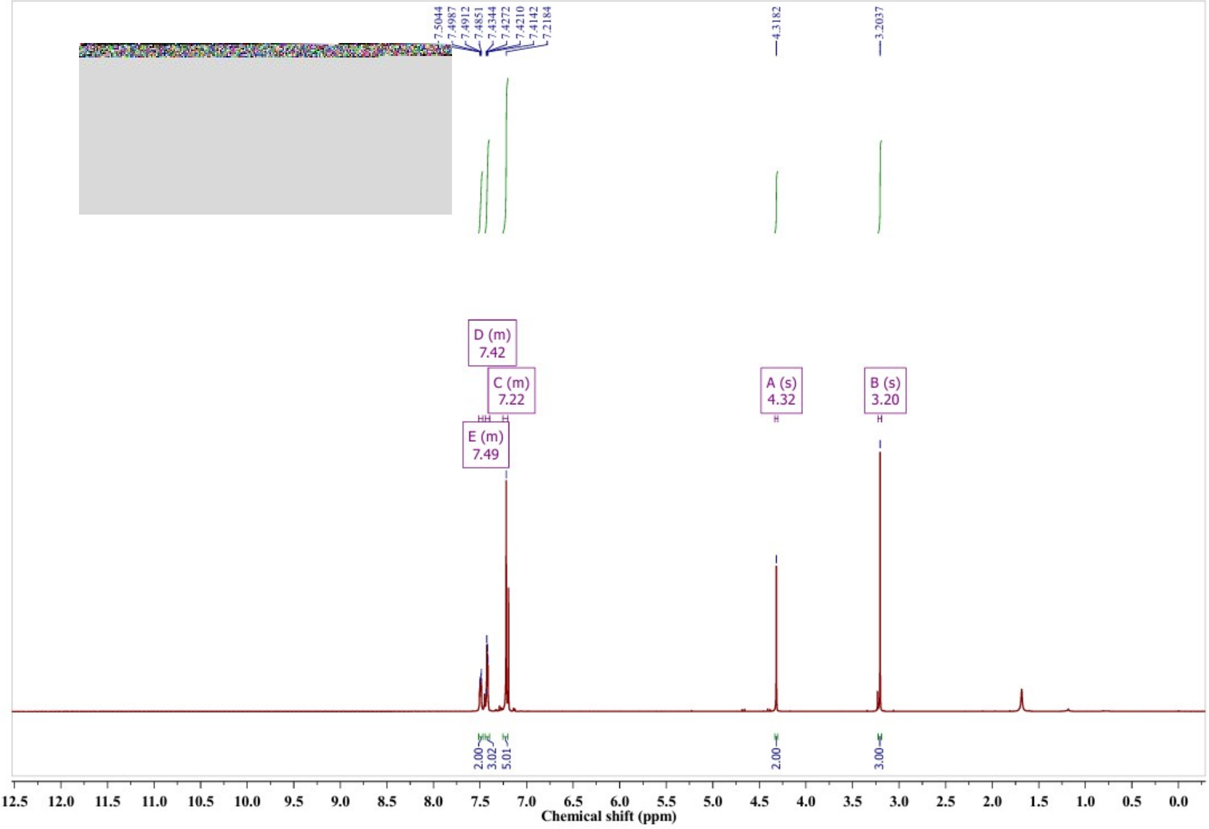

Figure S18 ^{1}H NMR spectrum of **CL1** in $CDCl_3$

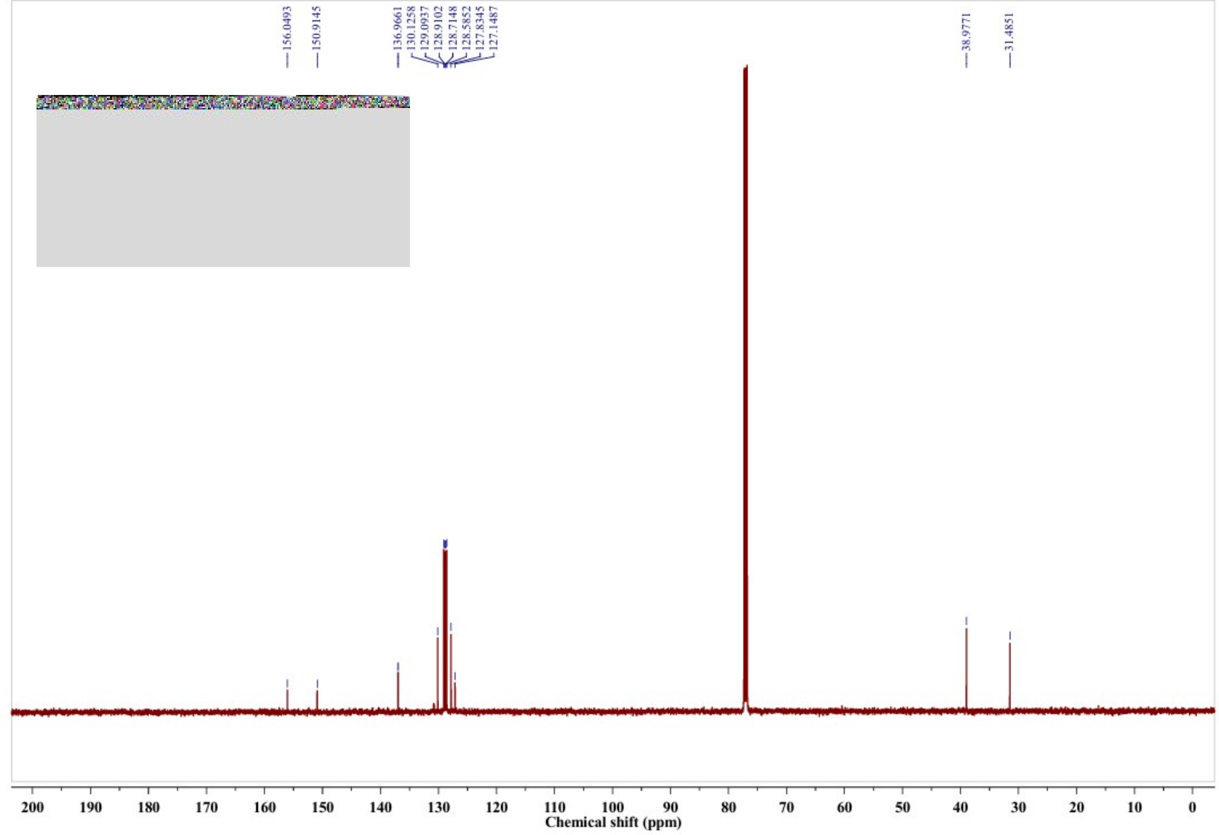

Figure S19 ^{13}C NMR spectrum of **CL1** in $CDCl_3$

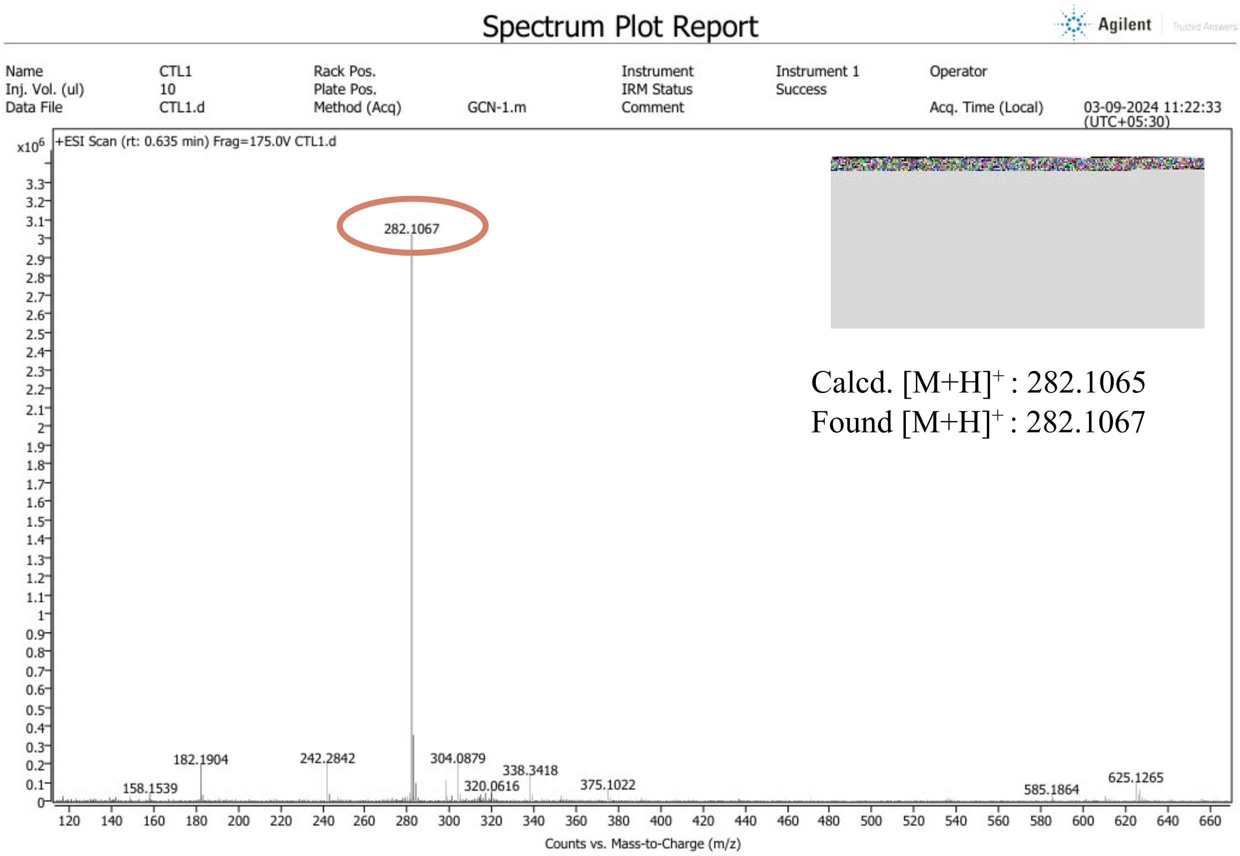

Figure S20 HRMS spectrum of **CL1**

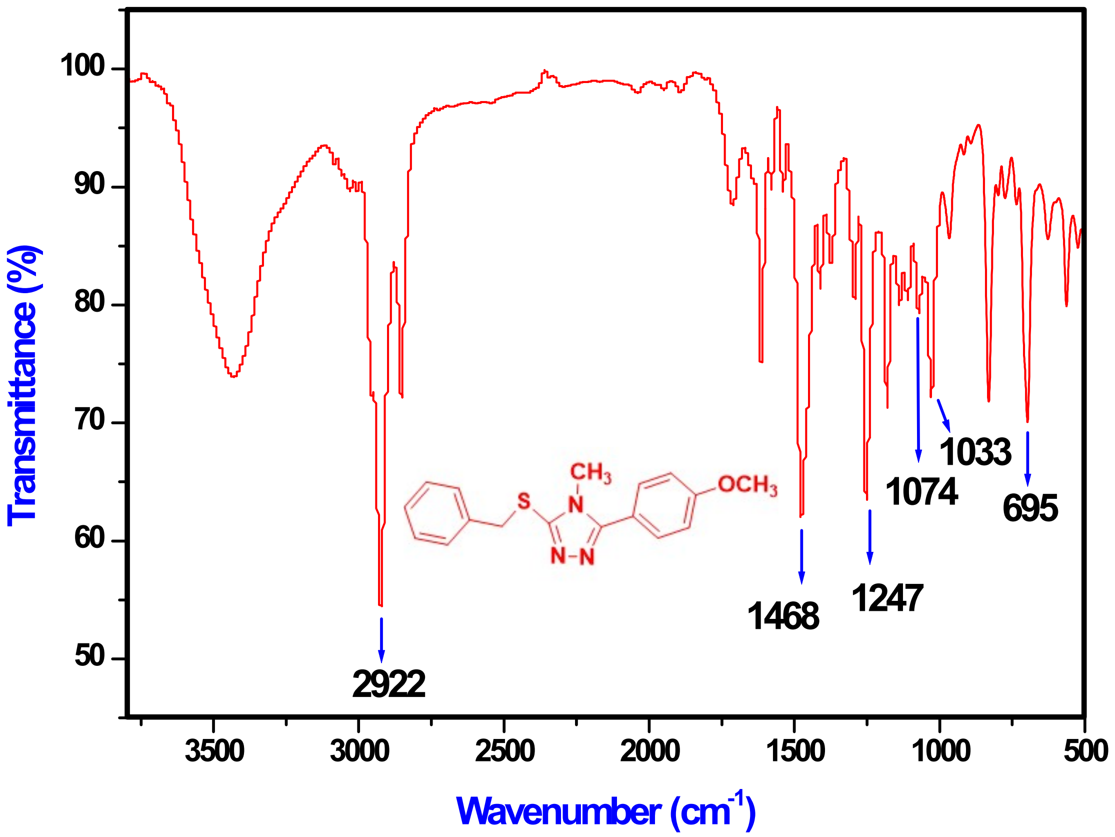

Figure S21 FT-IR spectrum of **CL2**

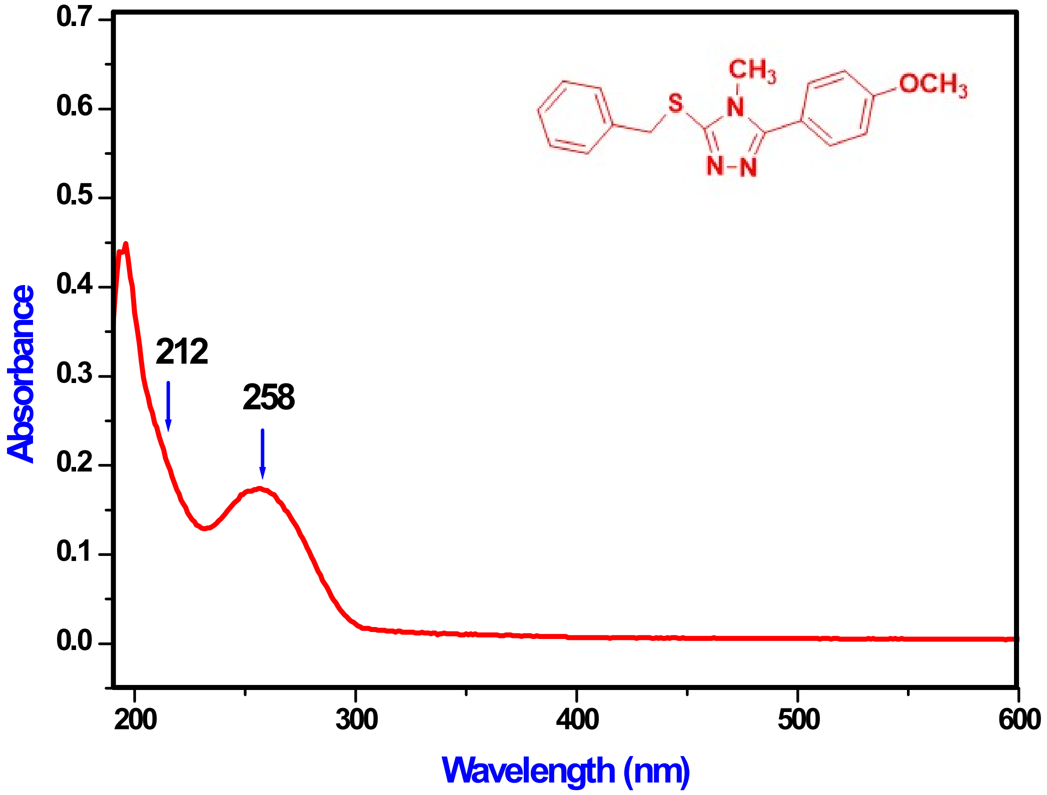

Figure S22 UV-Vis spectrum of **CL2**

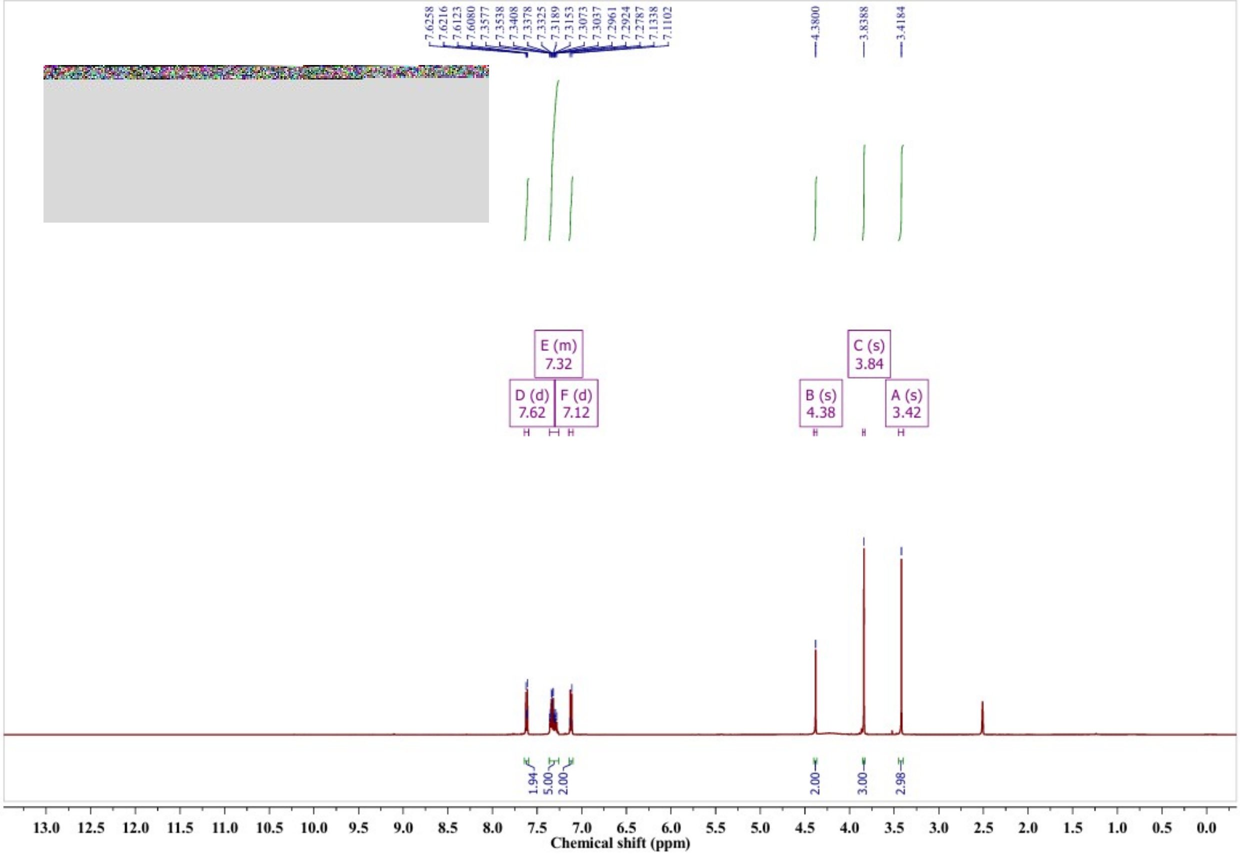

Figure S23 ^{1}H NMR spectrum of **CL2** in DMSO-d_6

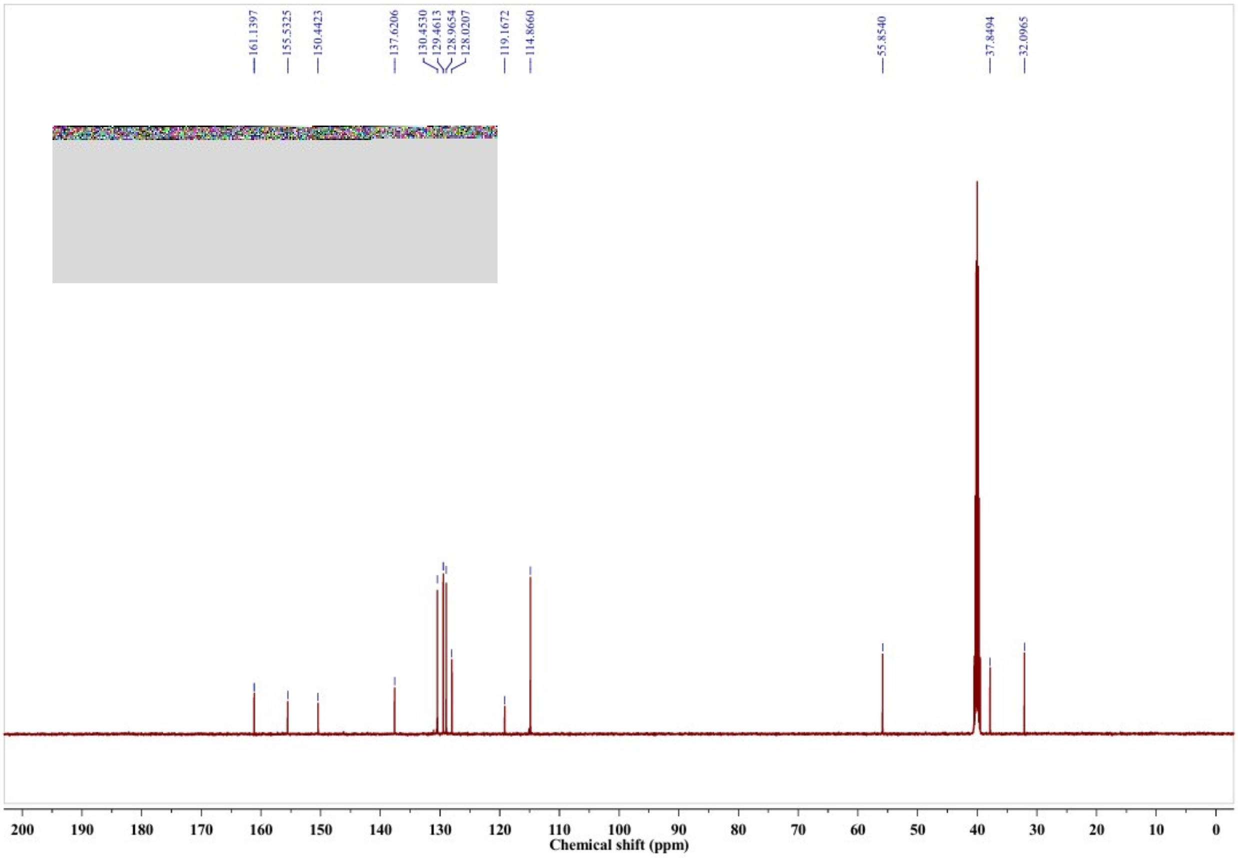

Figure S24 ^{13}C NMR spectrum of **CL2** in DMSO-d_6

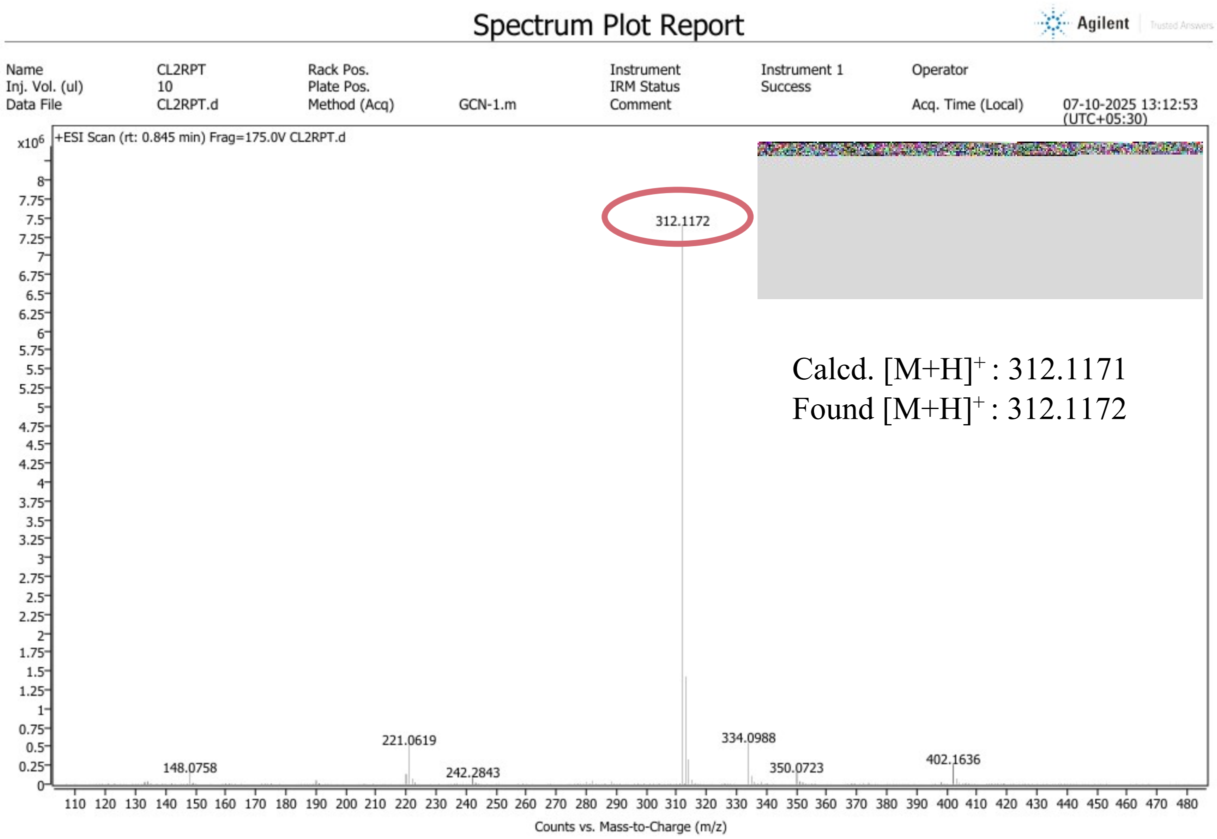

Figure S25 HRMS spectrum of **CL2**

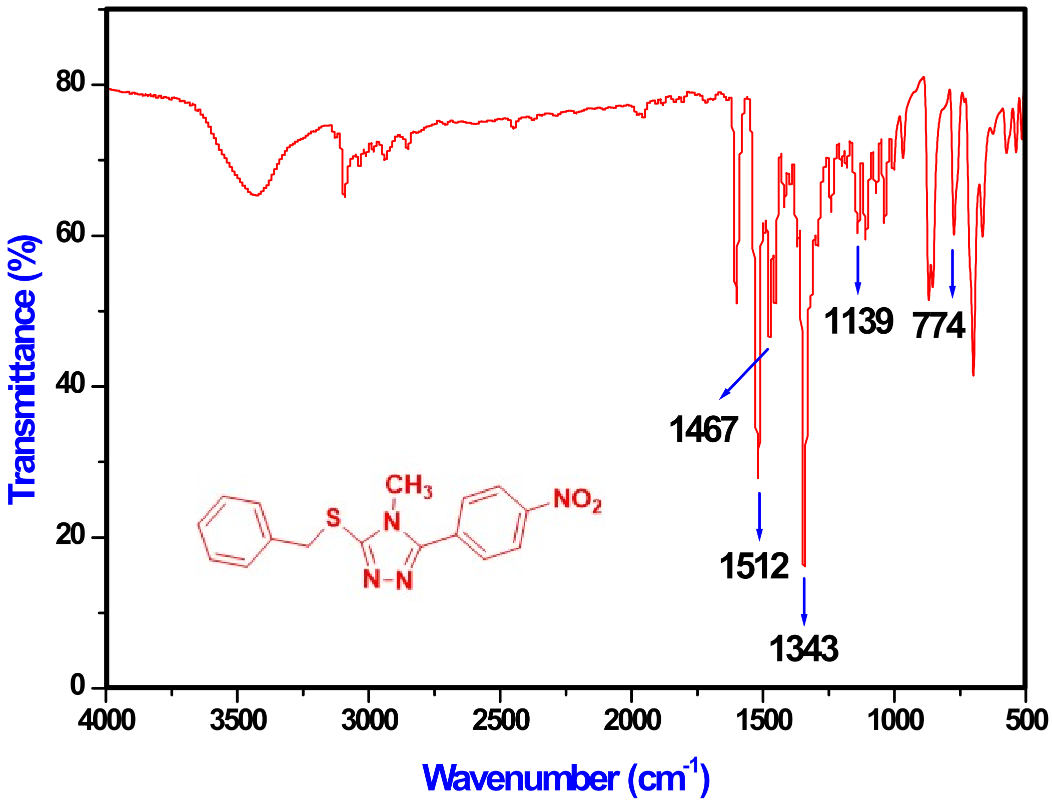

Figure S26 FT-IR spectrum of **CL3**

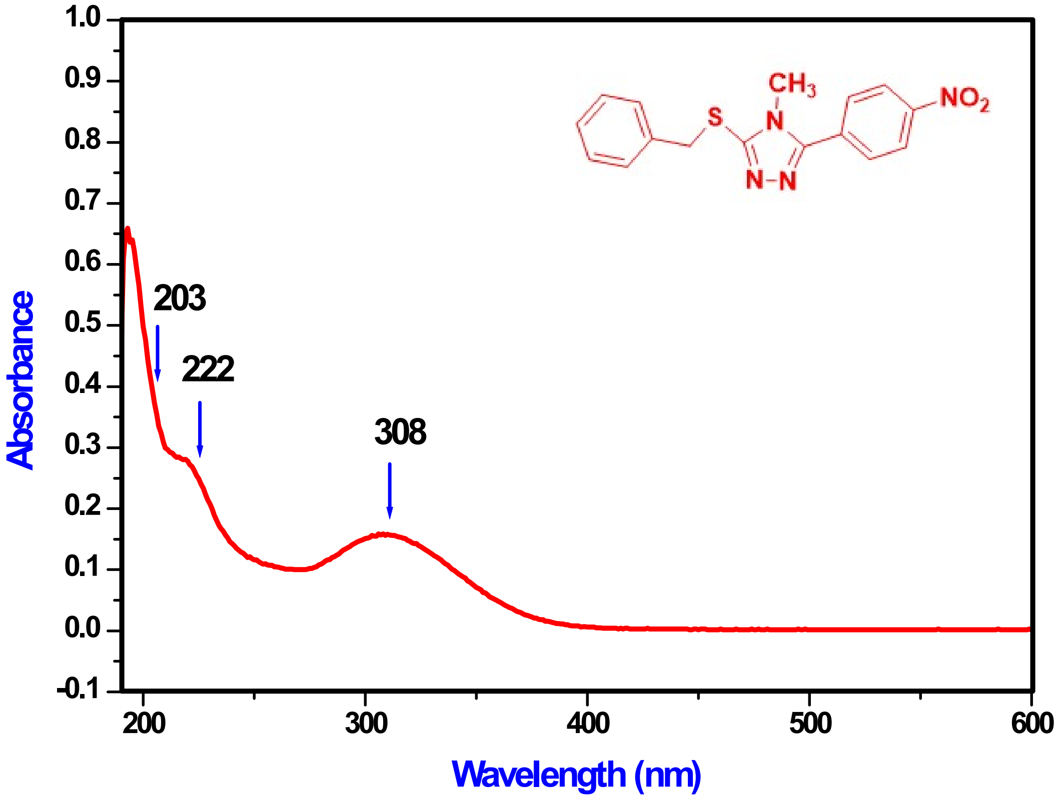

Figure S27 UV-Vis spectrum of **CL3**

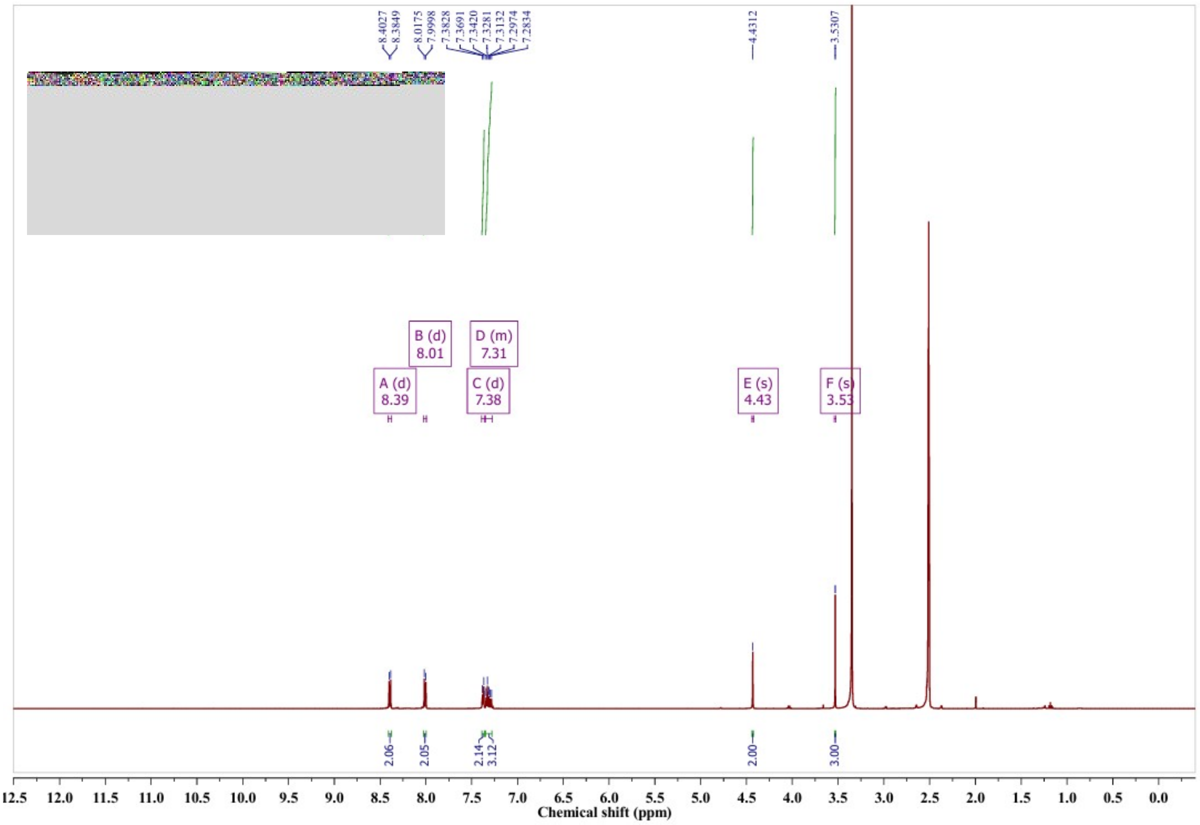

Figure S28 ^{1}H NMR spectrum of **CL3** in DMSO-d_6

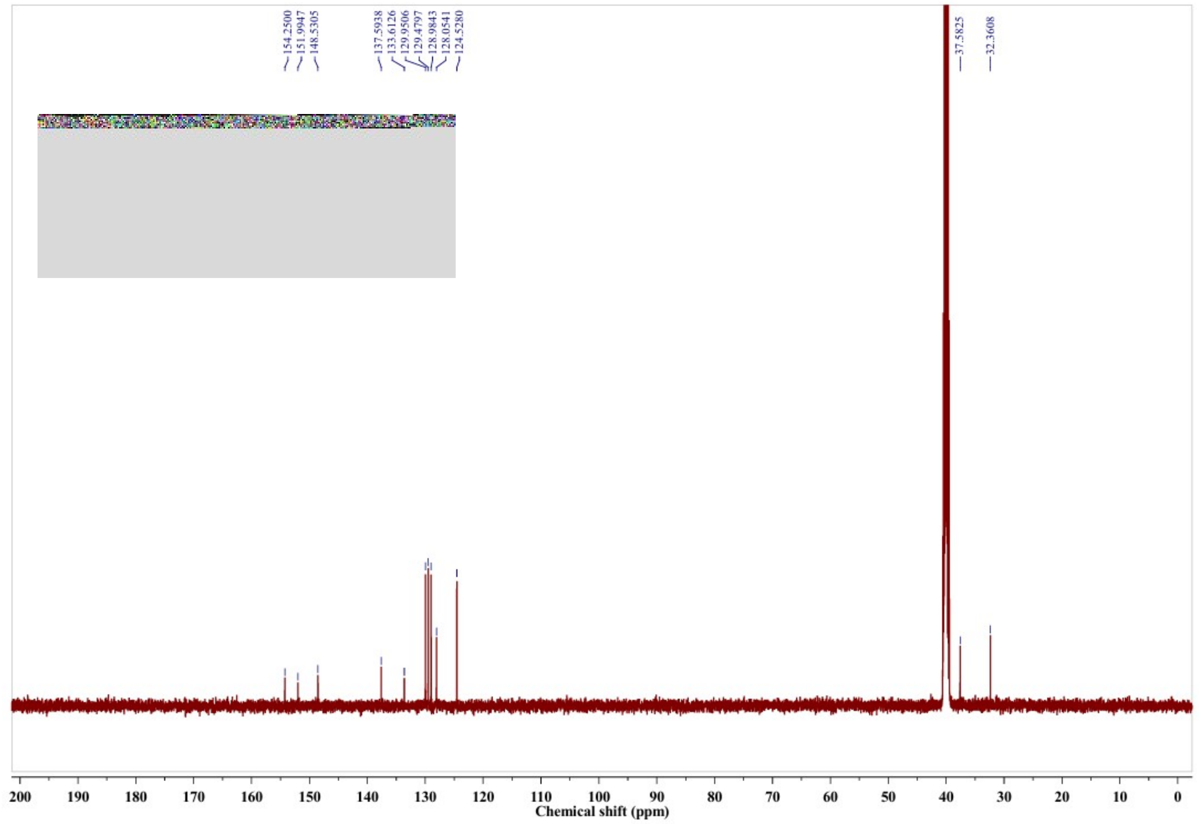

Figure S29 ^{13}C NMR spectrum of **CL3** in DMSO-d_6

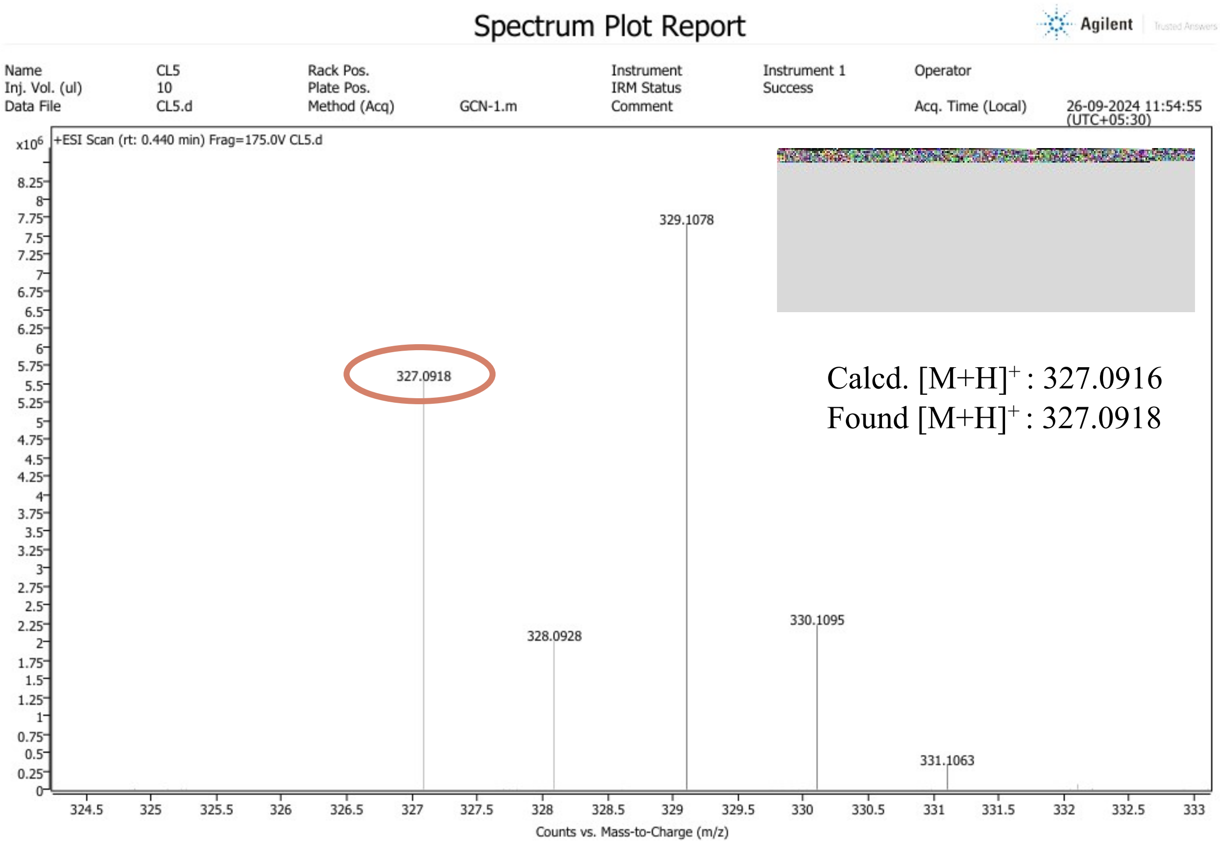

Figure S30 HRMS spectrum of **CL3**

Table S1 Crystallographic data and structure refinement details of isothiosemicarbazones

	**TL1**	**TL3**·HBr (*Z* isomer)	**TL3** (*E* isomer)
CCDC number	2506364	2506370	2506371
Formula	$C_{16}H_{17}N_3S$	$C_{16}H_{19}BrN_4O_3S$	$C_{16}H_{16}N_4O_2S$
$D_{calc.}$/g cm^{-3}	1.278	1.573	1.398
μ/mm^{-1}	1.884	4.393	1.979
Formula weight	283.38	427.32	328.39
Colour	yellow	yellow	orange
Shape	block-shaped	block-shaped	needle-shaped
Size/mm^3	0.32×0.23×0.08	0.22×0.09×0.05	0.50×0.05×0.04
T/K	100.00(10)	100.00(10)	100.00(10)
Crystal system	monoclinic	triclinic	monoclinic
Space group	$P2_1/c$	*P*-1	$P2_1$
a/Å	24.8331(2)	6.81290(10)	9.24200(10)
b/Å	11.78700(10)	9.15510(10)	4.94600(10)
c/Å	10.07740(10)	15.64500(10)	17.0656(2)
α/°	90	83.8980(10)	90
β/°	92.8390(10)	78.1950(10)	90.8380(10)
γ/°	90	71.0320(10)	90
V/Å^3	2946.11(5)	902.482(19)	780.00(2)
Z	8	2	2
Z'	2	1	1
Wavelength/Å	1.54184	1.54184	1.54184
Radiation type	Cu K_a	Cu K_a	Cu K_a
Θ_{min}/°	3.564	5.113	2.589
Θ_{max}/°	79.874	74.501	74.431
Measured reflections	21509	31293	14972
Independent reflections	6206	3698	2972
Reflections I≥2 s (I)	5643	3692	2933
R_{int}	0.0301	0.0487	0.0241
Parameters	363	228	209
Restraints	0	0	1
Largest peak	0.318	0.365	0.260
Deepest hole	-0.361	-0.500	-0.205
Goodness of Fit (GooF)	1.082	1.067	1.054
wR_2 (all data)	0.0968	0.0597	0.0642
wR_2	0.0946	0.0596	0.0638
R_1 (all data)	0.0377	0.0226	0.0239
R_1	0.0351	0.0225	0.0235

Table S2 Crystallographic data and structure refinement details of cyclized sulfanyl 1,2,4-triazole derivatives

	**CL1**·HBr	**CL2**	**CL3**
CCDC number	2506359	2506360	2506361
Formula	$C_{16}H_{16}N_3SBr$	$C_{17}H_{17}N_3OS$	$C_{16}H_{14}N_4O_2S$
$D_{calc.}$/g cm^{-3}	1.523	1.339	1.426
μ/mm^{-1}	4.743	1.899	2.030
Formula weight	362.29	311.39	326.37
Colour	colourless	colourless	yellow
Shape	block-shaped	block-shaped	block-shaped
Size/mm^3	0.19×0.15×0.07	0.21×0.10×0.06	0.17×0.06×0.02
T/K	99.98(10)	100.0(4)	100.00(10)
Crystal system	monoclinic	monoclinic	monoclinic
Space group	$P2_1/c$	$C2/c$	$P2_1/c$
a/Å	10.97500(10)	35.0245(3)	18.0524(4)
b/Å	13.76310(10)	8.18130(10)	7.4997(2)
c/Å	10.49770(10)	10.79360(10)	11.6915(2)
α/°	90	90	90
β/°	94.7090(10)	93.1190(10)	106.138(2)
γ/°	90	90	90
V/Å^3	1580.33(2)	3088.28(5)	1520.51(6)
Z	4	8	4
Z'	1	1	1
Wavelength/Å	1.54184	1.54184	1.54184
Radiation type	Cu K_a	Cu K_a	Cu K_a
Θ_{min}/°	4.042	2.527	2.548
Θ_{max}/°	74.497	80.135	74.492
Measured reflections	31861	32973	15216
Independent reflections	3223	3365	3116
Reflections I≥2 s (I)	3132	3175	2828
R_{int}	0.0521	0.0443	0.0306
Parameters	191	201	209
Restraints	0	0	0
Largest peak	0.855	0.239	1.398
Deepest hole	-1.021	-0.333	-0.472
Goodness of Fit (GooF)	1.074	1.108	1.050
wR_2 (all data)	0.0958	0.0982	0.1592
wR_2	0.0953	0.0972	0.1547
R_1 (all data)	0.0356	0.0381	0.0588
R_1	0.0351	0.0367	0.0549

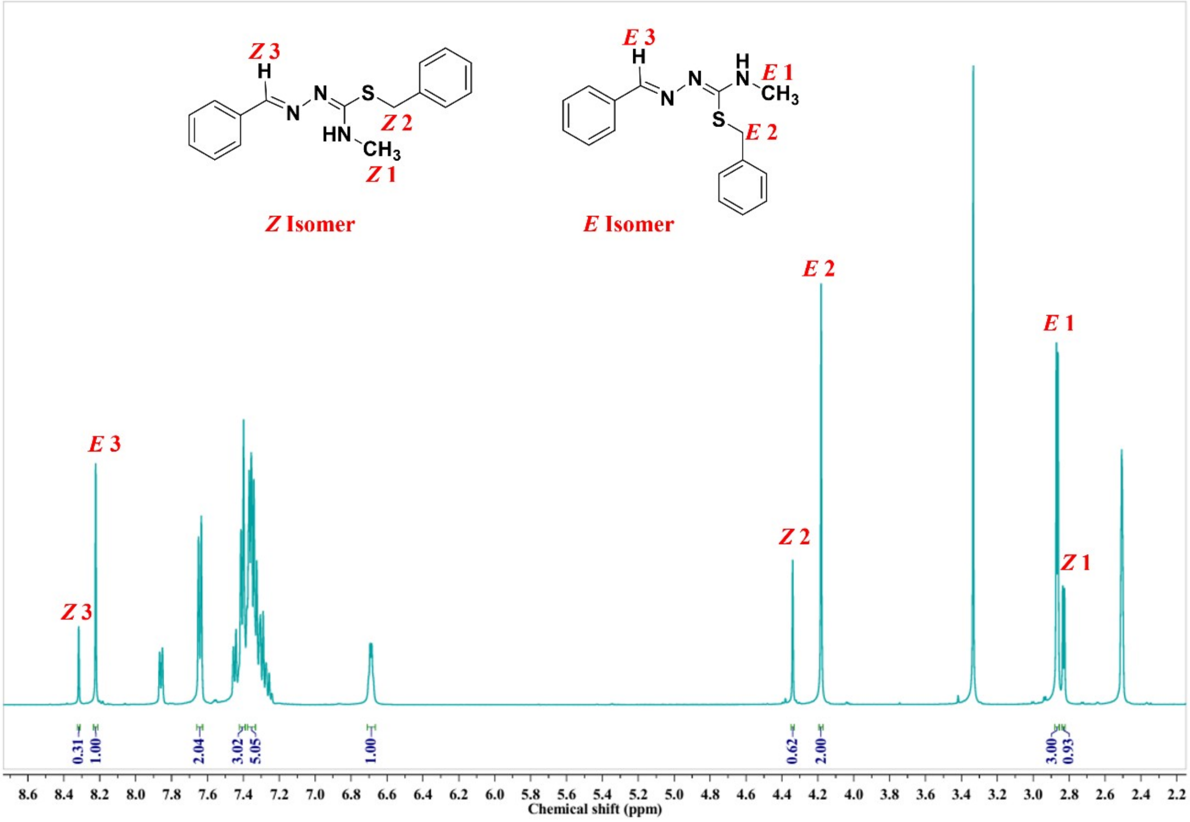

Figure S31 ^{1}H NMR spectrum of **TL1** (*E*/*Z* isomeric mixture) in DMSO-d_6

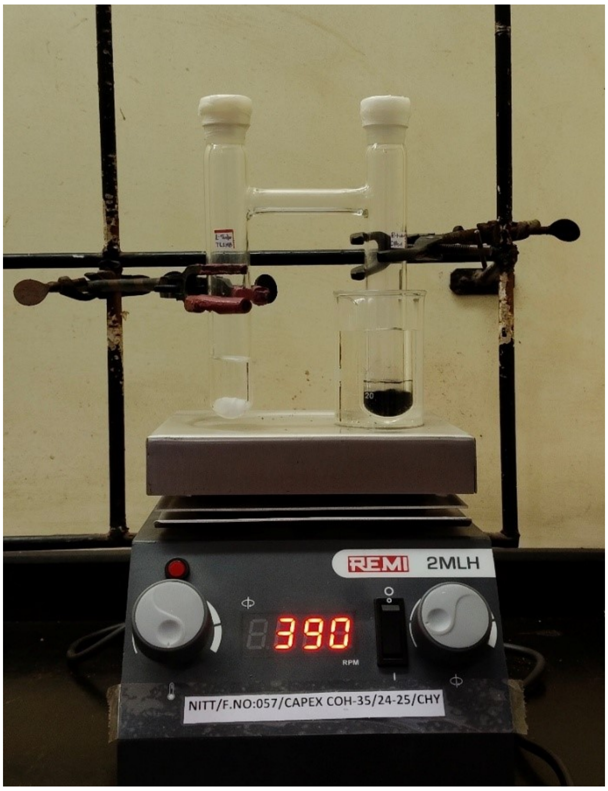

Figure S32 Experimental setup for the reduction of diphenylacetylene experiment

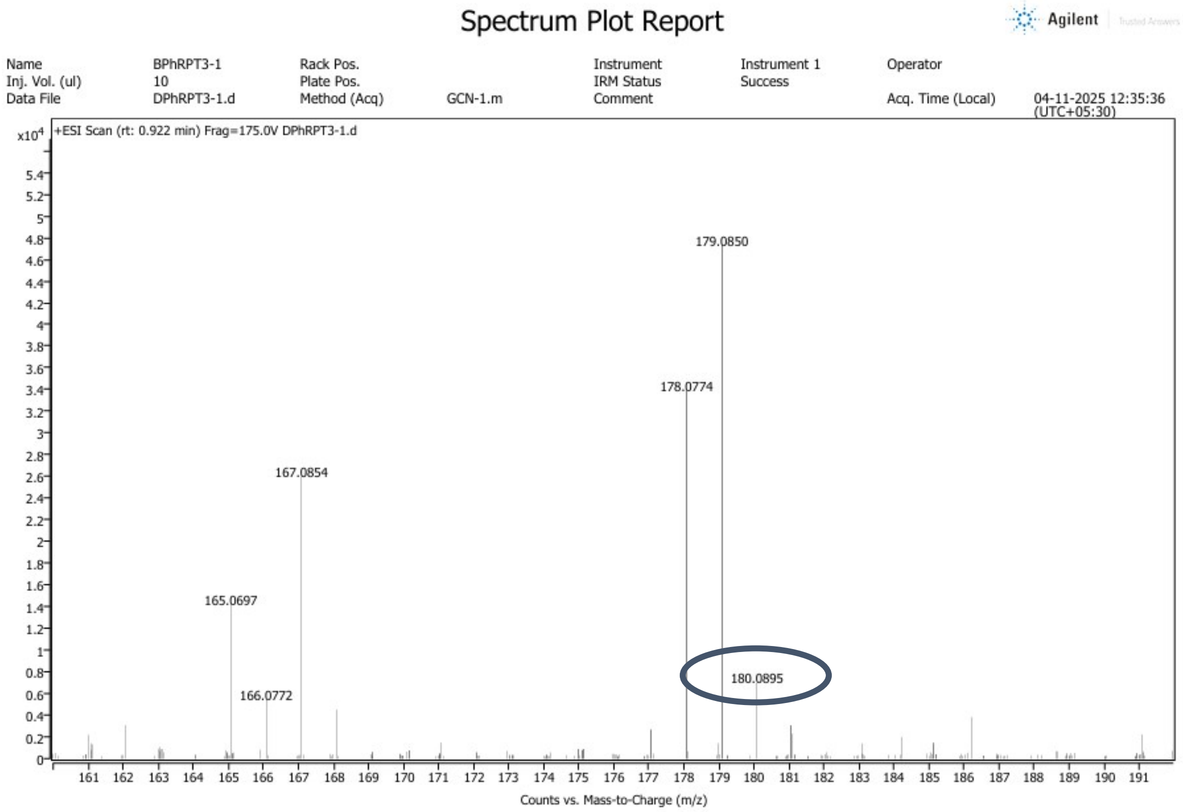

Figure S33 HRMS spectrum for the reduction of diphenylacetylene

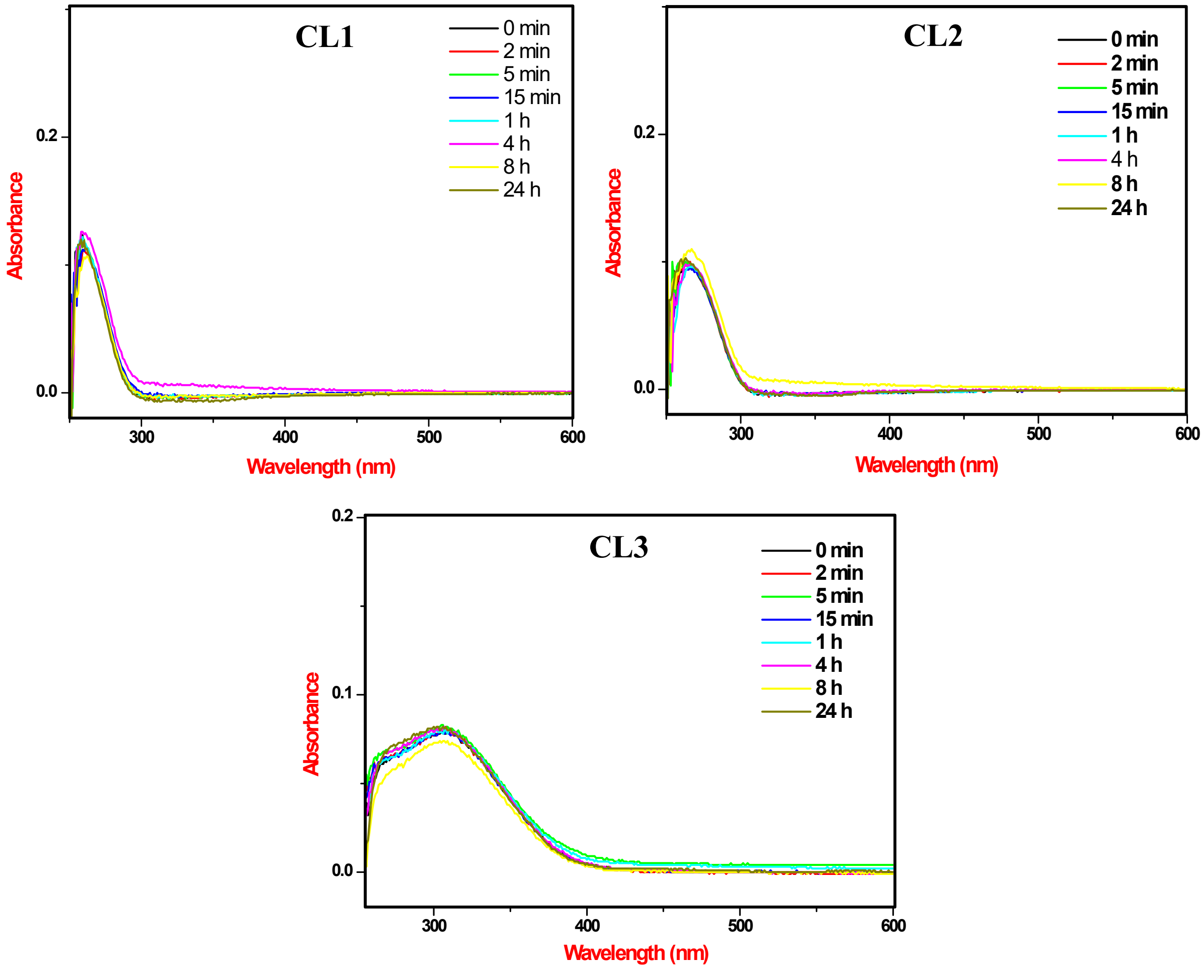

Figure S34 UV-Vis spectra of cyclized sulfanyl 1,2,4-triazole derivatives **CL1-CL3** in DMSO over a period of 24 h

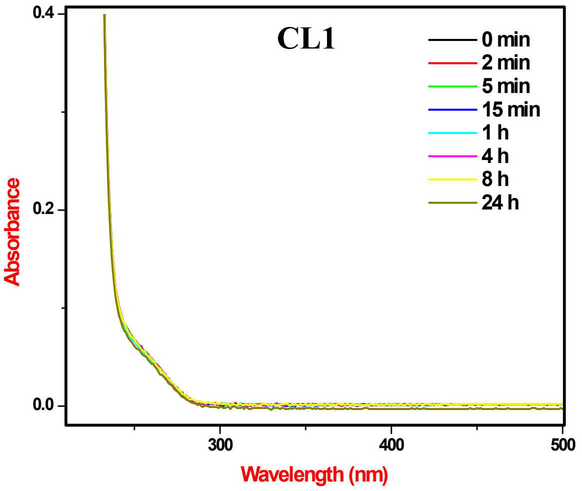

CL2

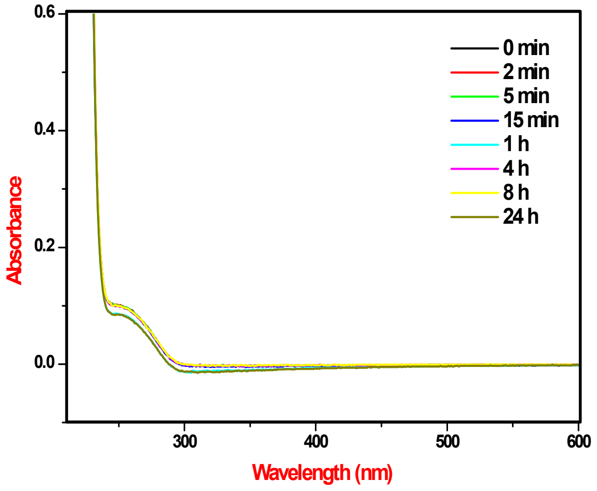

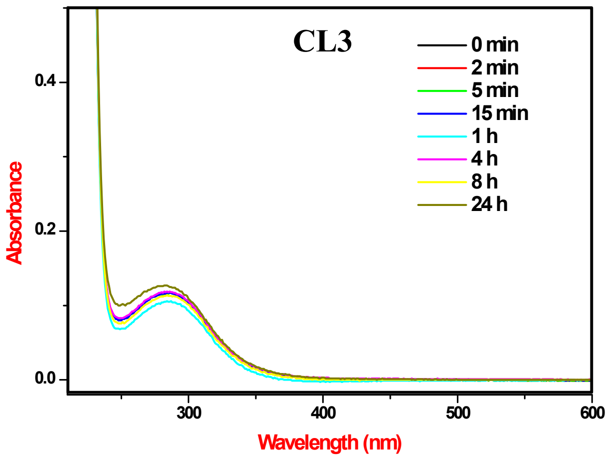

Figure S35 UV-Vis spectra of cyclized sulfanyl 1,2,4-triazole derivatives **CL1-CL3** in DMSO-water (1:99 v/v) over a period of 24 h

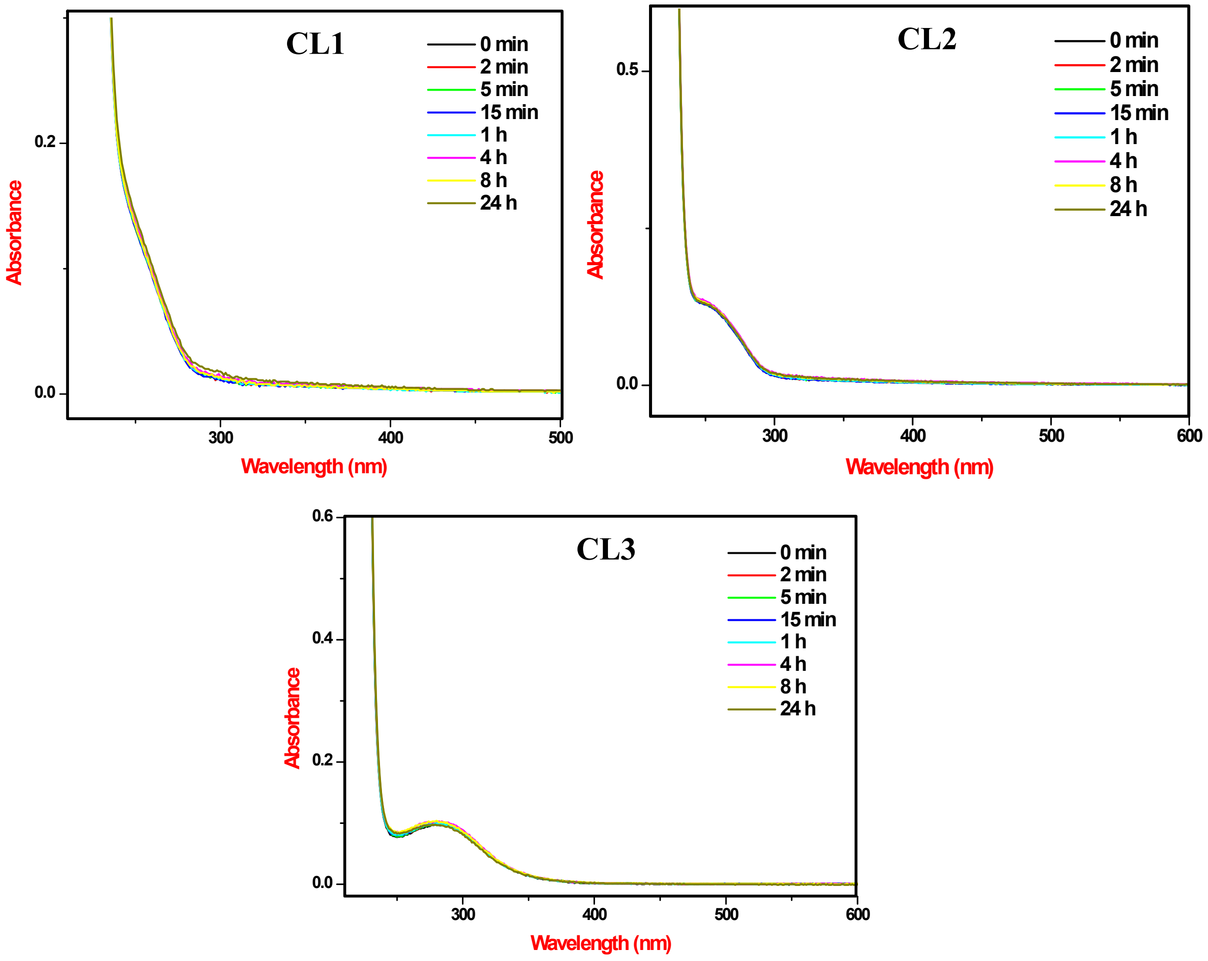

Figure S36 UV-Vis spectra of cyclized sulfanyl 1,2,4-triazole derivatives **CL1-CL3** in PBS (pH=7.4) over a period of 24 h
